# Interleukin-12 signaling drives Alzheimer’s disease pathology through disrupting neuronal and oligodendrocyte homeostasis

**DOI:** 10.1038/s43587-025-00816-2

**Published:** 2025-03-13

**Authors:** Shirin Schneeberger, Seung Joon Kim, Maria N. Geesdorf, Ekaterina Friebel, Pascale Eede, Marina Jendrach, Anastasiya Boltengagen, Caroline Braeuning, Torben Ruhwedel, Andreas J. Hülsmeier, Niclas Gimber, Marlene Foerster, Juliane Obst, Myrto Andreadou, Sarah Mundt, Jan Schmoranzer, Stefan Prokop, Wiebke Kessler, Tanja Kuhlmann, Wiebke Möbius, Klaus-Armin Nave, Thorsten Hornemann, Burkhard Becher, Julia M. Edgar, Nikos Karaiskos, Christine Kocks, Nikolaus Rajewsky, Frank L. Heppner

**Affiliations:** 1 Department of Neuropathology, Charité – Universitätsmedizin Berlin, corporate member of Freie Universität Berlin and Humboldt-Universität zu Berlin, Berlin, Germany; 2 Systems Biology of Gene Regulatory Elements, Berlin Institute for Medical Systems Biology (BIMSB), Max Delbrück Center for Molecular Medicine in the Helmholtz Association (MDC), Berlin, Germany; 3 Genomics Platform, Berlin Institute for Medical Systems Biology (BIMSB), Max Delbrück Center for Molecular Medicine in the Helmholtz Association (MDC), Berlin, Germany; 4 Department of Neurogenetics, Max Planck Institute for Multidisciplinary Sciences, Göttingen, Germany; 5 Department of Neurogenetics, Electron Microscopy Unit City Campus, Max Planck Institute for Multidisciplinary Sciences, Göttingen, Germany; 6 Institute of Clinical Chemistry, University of Zürich, Zürich, Switzerland; 7 AMBIO Advanced Medical Bioimaging Core Facility, Charité – Universitätsmedizin Berlin, Berlin, Germany; 8 Institute of Experimental Immunology, University of Zurich, Zurich, Switzerland; 9 Department of Pathology, College of Medicine, University of Florida, Gainesville, FL USA; 10 Evelyn F. and William L. McKnight Brain Institute, University of Florida, Gainesville, FL USA; 11 Center for Translational Research in Neurodegenerative Disease, University of Florida, Gainesville, FL USA; 12 Norman Fixel Institute for Neurological Diseases, University of Florida, Gainesville, FL USA; 13 Institute of Neuropathology, University Hospital Münster, Münster, Germany; 14 School of Infection and Immunity, University of Glasgow, Glasgow, UK; 15 Cluster of Excellence, NeuroCure, Berlin, Germany; 16 German Cancer Consortium (DKTK), Heidelberg, Germany; 17 German Center for Cardiovascular Research (DZHK), Berlin, Germany; 18 National Center for Tumor Diseases (NCT), Berlin, Germany; 19 Charité - Universitätsmedizin, Berlin, Germany; 20 German Center for Neurodegenerative Diseases (DZNE), Berlin, Germany

**Keywords:** Alzheimer's disease, Alzheimer's disease

## Abstract

Neuroinflammation including interleukin (IL)-12/IL-23-signaling is central to Alzheimer’s disease (AD) pathology. Inhibition of p40, a subunit of IL-12/IL-23, attenuates pathology in AD-like mice; however, its signaling mechanism and expression pattern remained elusive. Here we show that IL-12 receptors are predominantly expressed in neurons and oligodendrocytes in AD-like APPPS1 mice and in patients with AD, whereas IL-23 receptor transcripts are barely detectable. Consistently, deletion of the IL-12 receptor in neuroectodermal cells ameliorated AD pathology in APPPS1 mice, whereas removal of IL-23 receptors had no effect. Genetic ablation of IL-12 signaling alone reverted the loss of mature oligodendrocytes, restored myelin homeostasis, rescued the amyloid-β-dependent reduction of parvalbumin-positive interneurons and restored phagocytosis-related changes in microglia of APPPS1 mice. Furthermore, IL-12 protein expression was increased in human AD brains compared to healthy age-matched controls, and human oligodendrocyte-like cells responded profoundly to IL-12 stimulation. We conclude that oligodendroglial and neuronal IL-12 signaling, but not IL-23 signaling, are key in orchestrating AD-related neuroimmune crosstalk and that IL-12 represents an attractive therapeutic target in AD.

## Abstract (web summary)

Schneeberger, Kim, Geesdorf, Rajewsky, Heppner et al. tease IL-12 and IL-23 signaling apart in mouse models and human brain tissue to define a role for oligodendroglial and neuronal IL-12 signaling in neuroimmune crosstalk in Alzheimer’s disease.

## Main

**Fig. 1:**
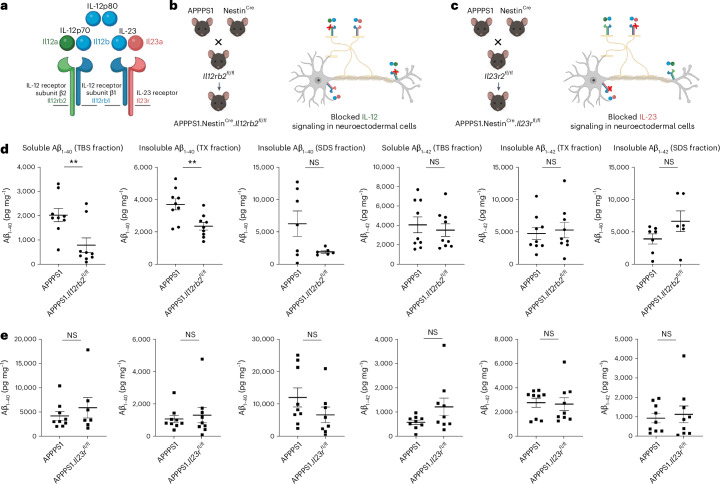
Deletion of IL-12-specific receptor subunit IL12Rβ2 results in reduction of amyloid burden. **a**, p40 can form monodimers (IL-12p80) or heterodimers (IL-12p70) consisting of p35 and p40. IL-12p70 binds to the dimerized IL-12Rβ2 and IL-12Rβ1. IL-23, consisting of p19 and p40, binds to the receptor subunits IL-23R and IL-12Rβ1. The genes that encode the respective protein subunits are shown in matched color. This illustration was created in BioRender: Geesdorf, M. (2025) https://BioRender.com/w20e262. **b**,**c**, By crossbreeding either *Il12rb2*^*fl/fl*^ or *Il23r*^*fl/fl*^ mice to APPPS1 and to Nestin^Cre^ animals, IL-12-specific or IL-23-specific receptor deletion was achieved in neuroectodermal cells of AD-like mice. This illustration was created in BioRender: Geesdorf, M. (2025) https://BioRender.com/m33j769 and https://BioRender.com/q27e982. **d**, Proteins from total brains of 250-day-old APPPS1.*Nestin*^*Cre*^*.Il12rb2*^*fl/fl*^ mice (*n* = 9) and APPPS1 littermates expressing functional *Il12rb2* (*n* = 9) were extracted based on their solubility and assessed for Aβ_1–40_ and Aβ_1–__42_ in the soluble (TBS) and insoluble (Triton-X and SDS) fractions using an electrochemiluminescence ELISA assay (Meso Scale). Aβ_1–40_: *t* = 3.062, df = 16, **P = 0.0075 for the TBS fraction; *t* = 3.256, df = 16, **P = 0.0050 for the TX fraction; and *t* = 2.034, df = 11, *P* = 0.668 for the SDS fraction; Aβ_1–42_: unpaired *t*-tests, *t* = 0.5092, df = 16, *P* = 0.6175 for the TBS fraction; *t* = 0.3554, df = 16, *P* = 0.7269 for the TX fraction; and *t* = 1.627, df = 11, *P* = 0.1319 for the SDS fraction. **e**, Aβ_1–40_ and Aβ_1–42_ levels in APPPS1.*Nestin*^*Cre*^*.Il23r*^*fl/fl*^ mice (*n* = 9) and APPPS1 littermates with functional IL-23 receptor subunit (*n* = 9) upon similar workup as described in **d**; Aβ_1–40_: *t* = 0.7989, df = 14, *P* = 0.4377 for the TBS fraction; *t* = 0.4474, df = 16, *P* = 0.6606 for the TX fraction; and *t* = 1.393, df = 15, *P* = 0.1838 for the SDS fraction; Aβ_1–42_: *t* = 1.710, df = 16, *P* = 0.1066 for the TBS fraction; *t* = 0.1808, df = 16, *P* = 0.8588 for the TX fraction; and *t* = 0.3960, df = 16, *P* = 0.69731 for the SDS fraction. Data were analyzed as two-tailed unpaired *t*-test; bars represent mean ± s.e.m. df, degrees of freedom; NS, not significant.

Pathological hallmarks of Alzheimer’s disease (AD) are the faulty aggregation and deposition of amyloid-β (Aβ) and tau proteins as well as pronounced neuroinflammation, which escalates with disease development. This process is primarily driven by microglia, the brain’s intrinsic myeloid cells. A key inflammatory pathway in AD pathology is interleukin (IL)-12 and IL-23 signaling. IL-12 levels are increased in brain tissue and the cerebral spinal fluid (CSF) of patients with AD and of patients with mild cognitive impairment (MCI)^1^. IL-12 and IL-23 are heterodimers comprising the subunits p35 or p19, respectively, while sharing the common subunit p40 (Il12b). p40 can also form homodimers and binds either as homodimer or heterodimer to the IL-12 receptor subunit β1 (Il12rb1) (Fig. 1a). IL-12-specific signaling is executed when, in combination with p40 actions, the IL-12 subunit p35 (Il12a) binds to the IL-12 receptor subunit β2 (Il12rb2), whereas IL-23-specific signaling is induced in concert with p40 when the IL-23 subunit p19 (Il23a) binds to the IL-23 receptor (Il23r)^2,3^. Each of these two IL-12 receptor subunits has been shown to occur as dimers/oligomers; the formation of these higher-order structures differing in their affinity to bind IL-12 is ligand independent, where IL-12R β2 expression appears to be crucial for regulating IL-12 responsiveness^4–6^. We showed previously that inhibition of p40, which is produced by microglia AD-specifically, resulted in a substantial reduction of AD-related pathology in transgenic mouse models of amyloidosis, including a reduction in amyloid deposition and reversal of cognitive deficits^7,8^. Although innate immune cells and T lymphocytes are well known to respond to IL-12 and IL-23 in a peripheral—that is, a non-central nervous system (CNS) inflammatory—setting^9^, IL-12/IL-23 actions within the amyloidogenic AD brain are not understood: neither the exact cellular players are known nor whether IL-12 and/or IL-23 individually, or in concert, confer this AD-specific effect.

## Results

### IL-12, not IL-23, is driving pathology in the amyloidogenic brain

To dissect whether IL-12 and/or IL-23 is driving the previously reported IL-12/IL-23/p40-mediated modulation of AD pathology in the amyloidogenic mouse brain^7^ on a functional level, we bred AD-like APPPS1 to mice in which either the IL-12-specific receptor or the IL-23-specific receptor can be deleted conditionally, namely *Il12rb2*^*fl/fl*^ or *Il23r*^*fl*/*fl*^ mice, resulting in APPPS1.*Il12rb2*^*fl/fl*^ or in APPPS1.*Il23r*^*fl*/*fl*^ mice. To identify those CNS cells that harbor the respective receptors, we crossed the aforementioned double transgenic mice to Nestin^Cre^ reporter mice, thus enabling excision of the floxed gene segment encoding either the IL-12 receptor or the IL-23 receptor, through Cre-mediated recombination in cells from the neuroectodermal lineage—that is, in astrocytes, oligodendrocytes and neurons (Fig. 1b,c). Measuring the amount of amyloid burden in solubility-based brain protein extracts, we observed a significant (biochemically Aβ_1–40_-dominant) reduction of Aβ burden in APPPS1.Nestin^Cre^.*Il12rb2*^*fl*/*fl*^ mice lacking the IL-12-specific receptor gene *Il12rb2* when compared to APPPS1 mice harboring the IL-12-specific receptor subunit β2 (Fig. 1d). In contrast, deleting IL-23 receptor in a similar fashion in neuroectodermal cells in APPPS1.Nestin^Cre^.*Il23r*^*fl*/*fl*^ mice did not result in changes in Aβ_1–40_ or Aβ_1–42_ in all protein fractions—that is, soluble TBS, insoluble Triton-X and SDS fractions contained Aβ_1–40_ and Aβ_1–42_ levels identical to APPPS1 littermates harboring the IL-23 receptor in the neuroectoderm (Fig. 1e). These experiments showed that exclusive deletion of IL-12 signaling, but not of IL-23, recapitulated the reduction by approximately 50% in Aβ burden in APPPS1.*Il12b*^−/−^ mice (lacking IL-12 and IL-23 in concert), as previously described by us^7^.

### Transcriptional regulation of IL-12/23 in the human CNS

**Extended Data Fig. 1:**
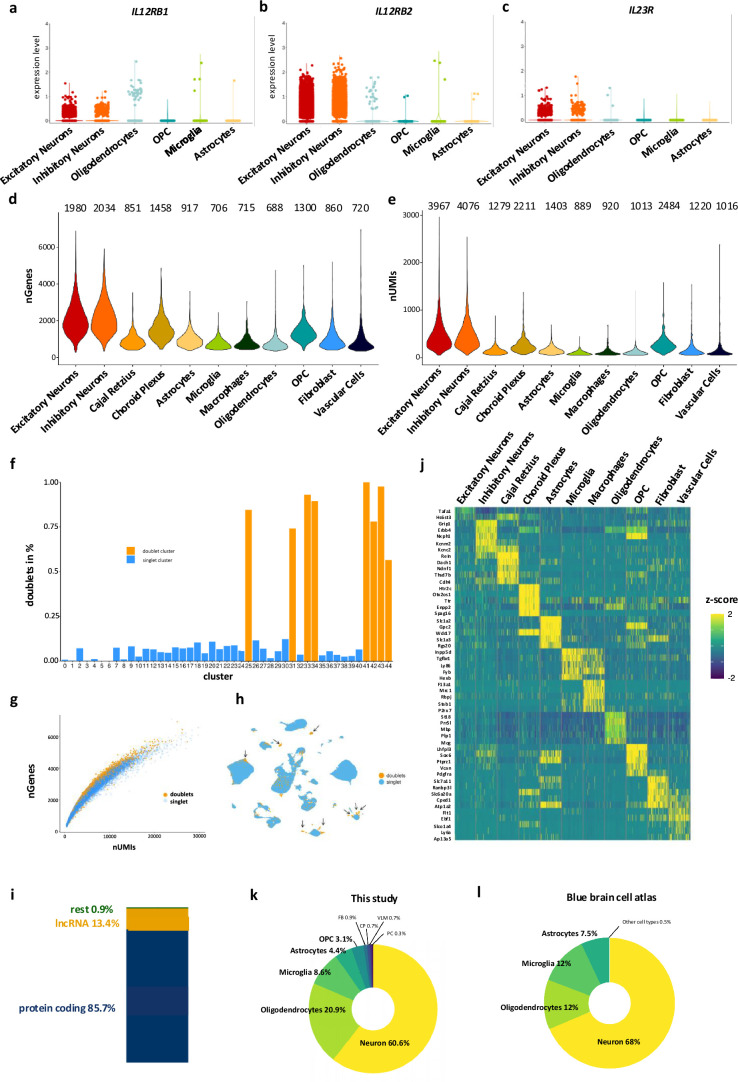
Human IL-12/IL-23 receptor expression and mouse snRNA-seq data quality and cluster annotation. Single-nucleus transcriptome data of 76,533 total nuclei isolated from two postmortem human primary motor cortex derived from portal.brainmap.org 4. **a-c**. Violin plots showing captured *IL12RB1*, *IL12RB2* and *IL23R* transcripts per cell type in the human cortex. **d,e**. Violin plots depicting various murine hippocampal cell types: gene count per cell (**d**) and UMI count per cell (**e**), averaging at 1,412 genes and 2,421 UMI, respectively. **f-h**. Doublet discrimination identified roughly 5% of all nuclei as doublets. From a total of 44 detected clusters, cluster 25, 31, 33, 34, 41, 42, 42 and 44 were flagged as clusters carrying doublets using Scrublet (v.0.21). Clusters with >50% of doublets were removed from further analysis. **i**. Bar graph depicting biotypes of detected RNA shows that approximately 85.7% of all transcripts were protein-coding followed by 13.4% long non-coding RNAs (lncRNAs). **j**. Heatmap showing cell type-specific lncRNAs. **k**. Cellular proportions of our snRNA-seq mouse study reflected the **l**. published cellular composition of the hippocampus according to the Blue Brain Cell Atlas.

To assess whether the molecular repertoire of IL-12/23 signaling is not only present in mice but also in the human CNS, and to bypass the lack of reliable detection tools, such as antibodies to most components of these multi-subunit cytokines and their receptors, we examined publicly available single-nucleus transcriptome datasets provided by the Allen Brain Map Atlas derived from postmortem human primary motor cortex tissue. Here, we found that *IL12RB1* and, even more strongly, *IL12RB2* transcripts were expressed by neurons and somewhat weaker by oligodendrocytes, whereas *IL23R* expression was rather faint^10^ (Extended Data Fig. 1a–c).

### Neurons and oligodendrocytes are IL-12 target cells

**Fig. 2:**
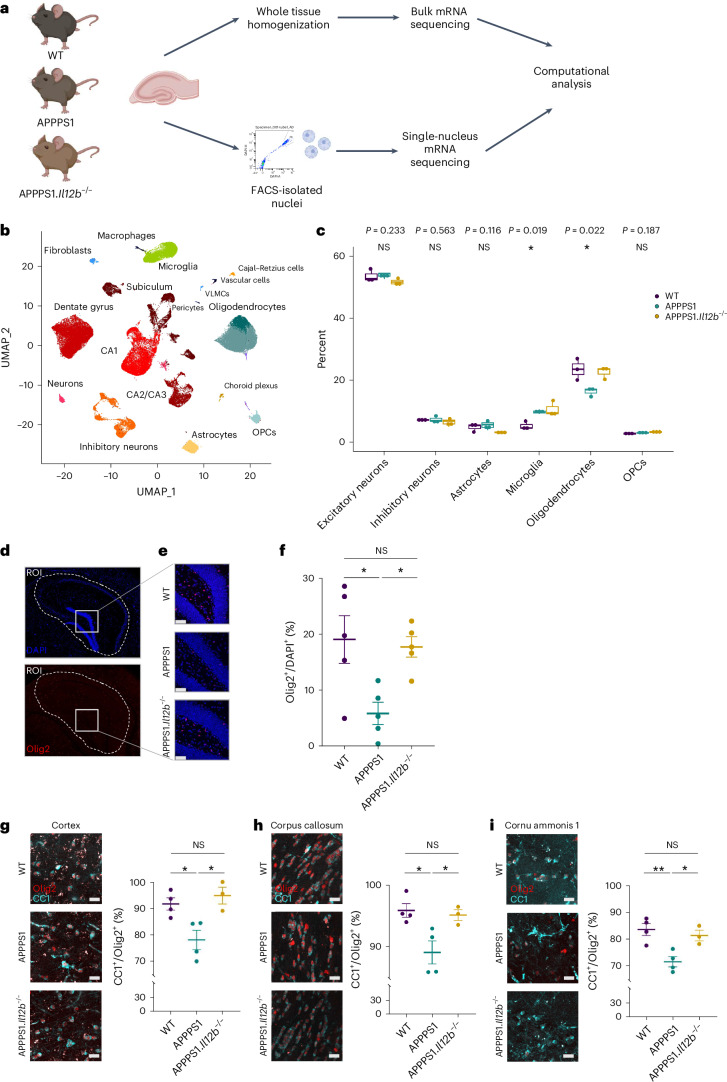
IL-12/Il-23 signaling reduces hippocampal oligodendrocytes in a mouse model of AD. **a**, Experimental outline. Isolated nuclei from hippocampi of 250-day-old animals (*n* = 3 per genotype), purified by FACS and used for snRNA-seq. Bulk RNA-seq libraries were prepared from RNA isolated of hippocampi (*n* = 3 per genotype). If not stated otherwise, figures reflect results from snRNA-seq. Illustration was created in BioRender: Geesdorf, M. (2025) https://BioRender.com/a60r345. **b**, UMAP plot showing 37 hippocampal cell clusters representing combined snRNA-seq data from three biological replicates per genotype. Cell types were assigned based on known markers. **c**, Cell type proportions in hippocampal samples across all three genotypes. Each dot represents one biological replicate (*n* = 3). One-way ANOVA with Holm–Bonferroni *P* value adjustment, df = 2. *F* = 1.945 for excitatory neurons, *F* = 0.563 for inhibitory neurons, *F* = 0.116 for astrocytes, *F* = 8.279 for microglia, *F* = 7.720 for oligodendrocytes and *F* = 2.245 for OPCs; boxplots show middle, median; lower hinge, 25% quantile; upper hinge, 75% quantile; upper/lower whisker, largest/smallest observation less/greater than or equal to upper/lower hinge ± 1.5× IQR. **d**, Mouse brain sections stained with DAPI (blue) and for Olig2 (red). The hippocampal outline was defined as the ROI (dashed white line) for quantifying Olig2^+^ cells. Scale bar, 100 µm. **e**, Representative zoomed-in images of brain tissue from WT, APPPS1 and APPPS1.*Il12b*^−/−^ mice showing Olig2^+^ cells. **f**, Quantification of Olig2^+^ cells normalized to DAPI^+^ cells in hippocampal regions (*n* = 5 per genotype with 3–6 sections per animal). One-way ANOVA with Tukey’s multiple comparison test, df = 2, *F* = 6.270, *P = 0.0137. Each symbol represents one mouse. Bars represent mean ± s.e.m. **g**–**i**, Quantification of CC1^+^/Olig2^+^ mature oligodendrocytes in cortex, corpus callosum and CA1. *n* = 4 for WT and APPPS1 and *n* = 3 for APPPS1.*Il12b*^−/−^ mice. Scale bar, 100 µm. **g**–**i**, One-way ANOVA with Tukey’s multiple comparison test on WT (mean = 91.67 ± s.e.m.), APPPS1 (mean = 77.99 ± s.e.m.) and APPPS1.*Il12b*^−/−^ (mean = 94.87 ± s.e.m.), df = 2, *F* = 0.8176, *P = 0.0116 (**g**); WT (mean = 95.85 ± s.e.m.), APPPS1 (mean = 89.05 ± s.e.m.) and APPPS1.*Il12b*^−/−^ (mean = 95.09 ± s.e.m.), df = 2, *F* = 7.132, *P = 0.0167 (**h**); WT (mean = 83.52 ± s.e.m.), APPPS1 (mean = 71.51 ± s.e.m.) and APPPS1.*Il12b*^−/−^ (mean = 81.31 ± s.e.m.), df = 2, *F* = 10.04, **P = 0.0066. (**i**). df, degrees of freedom; IQR, interquartile range; NS, not significant.

We next aimed to gain an unbiased understanding of IL-12 signaling in the amyloidogenic environment of the hippocampus, a brain area that is central for executing cognitive functions and known to be affected in AD pathology^11^. We characterized the transcriptional signature of individual hippocampi dissected from 250-day-old Aβ-overexpressing APPPS1 or APPPS1.*Il12b*^−/−^ as well as wild-type (WT) littermate control animals by single-nucleus RNA sequencing (snRNA-seq). Three independent experiments (Fig. 2a)—after removal of low-quality nuclei and doublets—yielded a total of 82,298 nuclei expressing an average of 1,412 genes and 2,421 transcripts (defined as unique molecular identifiers (UMIs)) (Extended Data Fig. 1d–h). Roughly 86% of all captured transcripts were protein-coding, whereas 13% comprised long non-coding RNAs (lncRNAs), many of which were cell type specific (Extended Data Fig. 1i–k). Cell types in the hippocampus encompassed excitatory and inhibitory neurons, Cajal Retzius cells, choroid plexus cells, astrocytes, microglia, macrophages, oligodendrocytes, oligodendrocyte progenitor cells (OPCs), fibroblasts and vascular cells. Doublets were identified in 5% of all nuclei and in eight of 44 clusters (Extended Data Fig. 1f–h), and those clusters harboring more than 50% of doublets were removed from further analysis.

**Extended Data Fig. 2:**
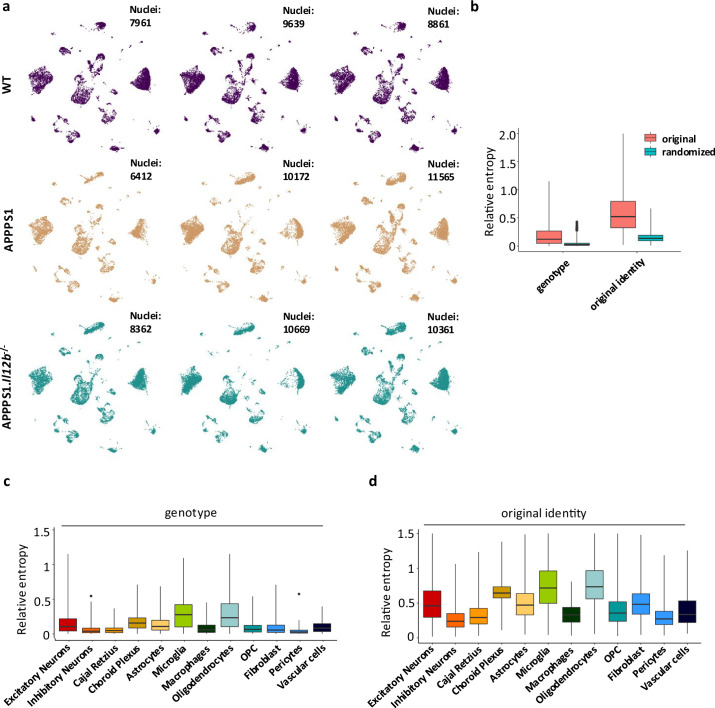
Absence of major batch effects in hippocampal snRNA-seq data from 250-day old mice. **a**. UMAP visualization of three individual biological replicates per genotype, showing mostly equal representations in each cluster. **b-d**. Entropy-based quantification of batch effects. The distribution of relative entropy values for cells of three individual biological replicates per genotype grouped by sample (original identity) or genotype was greater than randomizing these labels across cells (**b**). **c-d**. Different cell types grouped by genotype (**c**) and sample (**d**) (original identity); boxplots show: middle, median; lower hinge, 25% quantile; upper hinge, 75% quantile; upper/lower whisker, largest/smallest observation less/greater than or equal to upper/lower hinge ± 1.5 x IQR. Relative entropy per cell type was highest for cell types that differed biologically between genotypes, for example microglia and oligodendrocytes which react most strongly to amyloid and inflammatory conditions. Thus, differences were driven by biological meaningful distinctions rather than technical errors.

**Extended Data Fig. 3:**
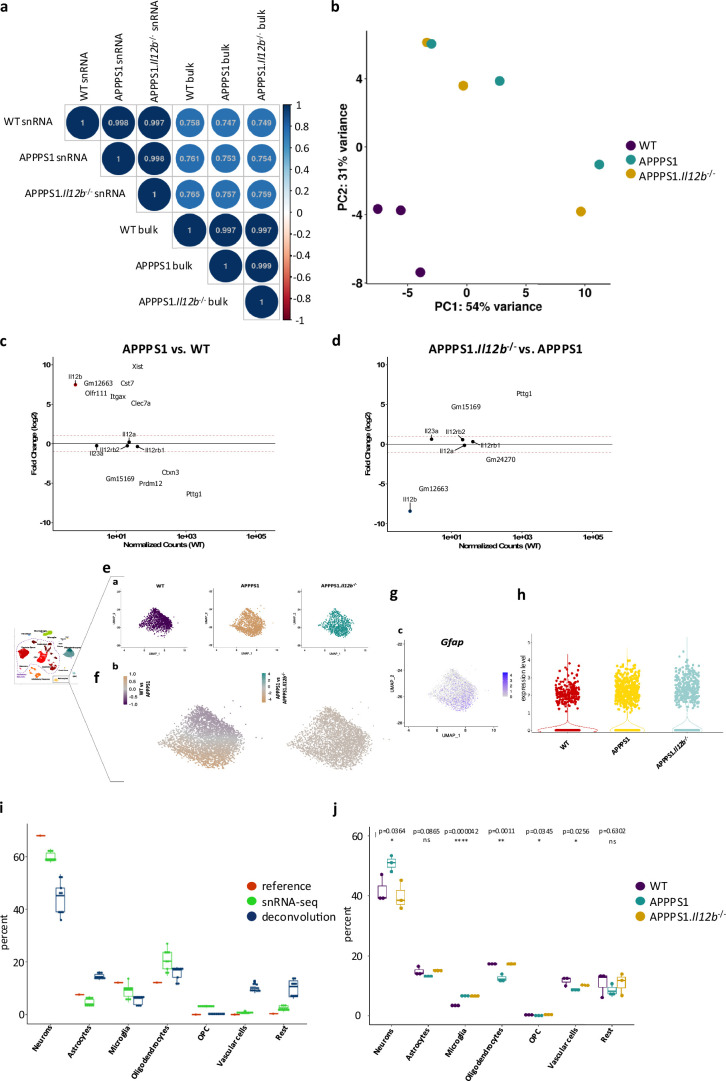
Various snRNA-seq and bulk RNA-seq analyses in 250-day-old mice reveal no significant changes in cell types other than oligodendrocytes and neurons. **a**. Correlation of hippocampal snRNA-seq and bulk RNA-seq data with respect to the ratio in appearance of distinct cell types (r ≥ 0.75). **b**. Principal component analysis of biological replicates (bulk RNA-seq), each replicate is represented by one dot. WT mice cluster together, while APPPS1 and APPPS1.*Il12b*^−/−^ mice are more scattered. **c-d**. MA plots comparing bulk gene expression of APPPS1 vs. WT mice (**c**) and of APPPS1.*Il12b*^*−/−*^ vs. APPPS1 mice (**d**). **e**. Close-up of the astrocyte gene signatures derived from single-nucleus RNA-seq of WT, APPPS1 and APPPS1.*Il12b*^−/−^ mice. **f**. Density plots illustrating the distribution of overall captured transcripts in WT vs. APPPS1 and APPPS1 vs. APPPS1.*Il12b*^−/−^ mice. **g**. Feature plot of the distribution of *Gfap* transcripts in the astrocyte cluster and **h**. *Gfap* expression levels in WT, APPPS1 and APPPS1.*Il12b*^*−/−*^. **i**. Comparison of cellular proportions of three biological replicates per genotype determined by three independent methods, namely the previously published reference data set (red), snRNA-seq (green) and deconvolution of bulk RNA-seq data (dark blue). Of note, the deconvolution matched the other two reference data sets best in OPCs, microglia and oligodendrocytes. Boxplots show: middle, median; lower hinge, 25% quantile; 45 upper hinge, 75% quantile; upper/lower whisker, largest/smallest observation less/greater than or equal to upper/lower hinge ± 1.5 x IQR. **j**. Cellular proportions gathered from deconvoluted bulk RNA-seq data across all three genotypes with three biological replicates each showed enhanced numbers of microglia in APPPS1 and APPPS1.*Il12b*^−/−^ mice and a rescue of oligodendrocytes numbers in APPPS1.*Il12b*^−/−^ mice. All df = 2, F = 6.053 for neurons, F = 3.783 for astrocytes, F = 83.238 for microglia, F = 26.317 for oligodendrocytes, F = 6.216 for OPCs, F = 7.182 for vascular cells, F = 0.499 for rest. One-way ANOVA with Holm-Bonferroni *P* value adjustment; boxplots show: middle, median; lower hinge, 25% quantile; upper hinge, 75% quantile; upper/lower whisker, largest/smallest observation less/greater than or equal to upper/lower hinge ± 1.5 x IQR.

Unsupervised clustering followed by uniform manifold approximation and projection (UMAP) for visualization revealed 37 clusters that were assigned to various neuronal, glial and other cell types based on the expression of known marker genes (Fig. 2b and Extended Data Fig. 1j)^12,13^. We identified 18 clusters of excitatory neurons, which were assigned to the anatomical region of the dentate gyrus, the cornu ammonis (CA) 1, CA2/CA3 and the subiculum; three clusters of inhibitory neurons; seven clusters of glial cells (microglia, astrocytes, oligodendrocytes and OPCs); and a small cluster with features of Cajal Retzius cells. Non-neural cells comprised myeloid cells, such as peripheral macrophages (distinct from CNS-resident microglia) and small clusters of fibroblasts and choroid plexus and endothelial cells. The hippocampus as a particularly neuron-rich brain region revealed a majority of neurons among the recovered nuclei (60% combined of excitatory and inhibitory neurons), followed by glia (37% combining microglia, oligodendrocytes, astrocytes and OPCs) (Extended Data Fig. 1k). These cell type proportions are consistent with the cell type composition of the mouse hippocampus—for example, as determined by the Blue Brain Cell Atlas (68% neuronal versus 32% of glial cells) (Extended Data Fig. 1l)^14,15^. The snRNA-seq data from all three independent biological replicates were superimposable—that is, showed no major batch effects and justified data aggregation without resorting to batch correction or alignment procedures (Extended Data Fig. 2a–d). This indicates that an *n* = 3 per genotype in our hands is a sufficient group size for downstream analyses. Furthermore, gene expression levels correlated between hippocampal snRNA-seq and bulk RNA-seq data (*R* ≥ 0.75), indicating that the single-nucleus data reflect the transcript composition of the intact tissue (Extended Data Fig. 3a).

A major pathological hallmark of AD is a substantial phenotypic alteration and proliferation of CNS-resident microglia and astrocytes. This is reflected by distinct transcriptome profiles of WT versus APPPS1 mice including an AD-specific upregulation of microglial pro-inflammatory genes, such as *Il12b* and *Clec7a* (Extended Data Fig. 3b,c)—a phenotype that was largely reverted upon deleting *Il12b* in APPPS1 mice (Extended Data Fig. 3d). Close-ups of the astrocyte populations in both AD-related genotypes, namely in APPPS1 and APPPS1.*Il12b*^−/−^ mice, showed an equally Aβ-reactive, *Gfap*-enriched inflammatory astrocyte cluster, suggesting that the lack or presence of p40 does not alter the astrocytic phenotype in the amyloidogenic CNS (Extended Data Fig. 3e–h). Interestingly, we observed a substantial reduction of oligodendrocytes, but not of their progenitors, in APPPS1 mice compared to WT mice (Fig. 2c). Notably, the AD-specific decrease in oligodendrocytes was rescued in APPPS1.*Il12b*^−/−^ mice. Cell type deconvolution of bulk RNA-seq data^16^ and quantification of oligodendrocytes in brain tissue sections by means of immunohistochemistry confirmed these findings (Fig. 2d–f and Extended Data Fig. 3i,j). Olig2^+^ cells were significantly reduced in the hippocampus of APPPS1 mice, and this reduction was reversed to the WT level in APPPS1.*Il12b*^−/−^ mice (Fig. 2f). We further quantified CC1^+^/Olig2^+^ mature oligodendrocytes in the hippocampus, cortex and corpus callosum of APPPS1, WT and APPPS1.*Il12b*^−/−^ mice. In each of these areas, mature CC1^+^/Olig2^+^ oligodendrocytes were significantly decreased in APPPS1 versus WT mice, whereas they were unaltered compared to WT in APPPS1.*Il12b*^−/−^ mice (Fig. 2g–i). In contrast, overall Olig2^+^ cells were not substantially changed in numbers in the cortex and corpus callosum of these mice. These data speak in favor of an IL-12-dependent loss of mature oligodendrocytes in the amyloid-rich brain, which can be rescued by interfering with IL-12 signaling.

**Fig. 3:**
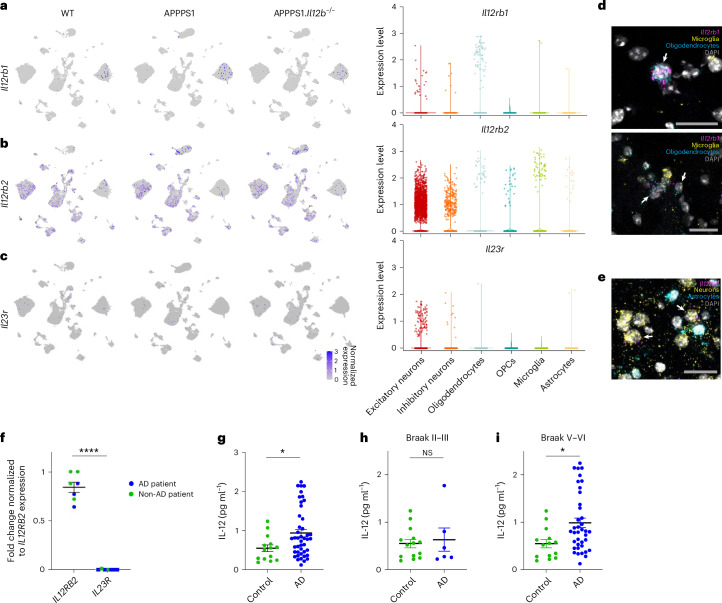
IL-12 and IL-23 receptor transcript expression and IL-12 protein levels in mouse and/or human postmortem brain tissue. **a**, *Il12rb1*, coding for IL-12 receptor subunit β1, is equally expressed across all three mouse genotypes (as indicated) and occurs most pronouncedly in oligodendrocytes. Violin plot showing captured *Il12rb1* transcripts across cell types. **b**, *Il12rb2*, coding for IL-12 receptor subunit β2, is strongly expressed in neurons and, to a lesser extent, in microglia, oligodendrocytes, OPCs and astrocytes. Violin plot showing captured *Il12rb2* transcripts across cell types. **c**, *Il23r* transcripts were only barely expressed in the aged mouse hippocampus. Violin plot showing captured *Il23r* transcripts across cell types. **d**, smFISH on brain tissue of APPPS1 mice revealed *Il12rb1* mRNA^*+*^ puncta (pink) in oligodendrocytes (marked by expression of *Sox10* mRNAs (blue)); microglia expressing *Tmem119* and *Sall1* are marked by yellow puncta; DAPI shown in gray depicts cell nuclei. **e**, *Il12rb2* mRNA^*+*^ puncta (pink) in neurons (marked by *Map2* and *NeuN* mRNAs (yellow)); astrocytes expressing *Aldh1l1*, *Gfap* and *Glast* are marked in blue; DAPI shown in gray depicts cell nuclei. Signals specific to *Il23r* mRNA were not detectable. Scale bar, 25 μm. **f**, Human postmortem hippocampal brain tissue from individuals without dementia (*n* = 4) and from patients with AD (*n* = 3). qPCR results showing *IL12RB2* and *IL23R* gene expression in bulk human hippocampal tissue, ****P = 1.678 × 10^−9^, *t* = 16.14, df = 12. Statistical analysis using two-tailed unpaired Student’s *t*-test. **g**–**i**, IL-12p70 protein as measured by ELISA in soluble tissue fraction of frontal cortex from age-matched non-AD controls (*n* = 14) and from patients with AD (*n* = 44). Statistical analysis using two-sided Mann–Whitney test for age-matched healthy controls (median = 0.5395 ± s.e.m.) and for patients with AD (median = 0.7946 ± s.e.m.), *P = 0.0316; Braak II–III: *P* = 0.8411, Braak V–VI: *P = 0.0117. df, degrees of freedom; NS, not significant.

To assess transcript expression of both IL-12 receptor subunits and of IL-23 receptor particularly in neurons and oligodendrocytes, we determined the cell-type-specific expression of IL-12/IL-23-associated transcripts within our snRNA-seq data. *Il12rb1* was mostly expressed in oligodendrocytes (Fig. 3a), whereas *Il12rb2* was more generally distributed across several cell types while clearly pronounced in neurons and oligodendrocytes (Fig. 3b). Transcripts encoding the IL-23 receptor (*Il23r*) were barely detectable in the hippocampus and absent in oligodendrocytes (Fig. 3c), suggesting that IL-23 is not involved in mediating the p40-dependent changes described in APPPS1 mice^7^. To visualize transcript expression, we applied single-molecule RNA fluorescence in situ hybridization (smFISH) on aged mouse brain tissue, where each fluorescent spot corresponds to one single RNA transcript. In line with our snRNA-seq data (Fig. 3a), we found *Il12rb1* transcripts in *Sox10*^*+*^oligodendrocytes and *Il12rb2* transcripts in *Rbfox3*^*+*^/*Tubb3*^*+*^neurons, whereas *Il23r* signals were not detectable in either cell type (Fig. 3d,e).

qRT–PCR of postmortem hippocampal brain tissue from non-demented controls or patients with AD (Fig. 3f and Supplementary Data Table 1) corroborated the mouse and human data: irrespective of AD pathology, we found *IL12RB2* transcripts specific for the IL-12 receptor to be expressed in human brains, whereas transcripts for the IL-23 receptor were lacking. As the receptor expression abundance did not change with disease status, we next wondered whether the ligand IL-12p70 does. Using human postmortem brain tissue of 44 AD cases and 14 age-matched non-demented control cases, we found IL-12p70 protein to be elevated in AD brains (Fig. 3g). Of note, IL-12p70 protein expression was mainly pronounced in AD cases with higher Braak stages (Braak stages V–VI) (Fig. 3h,i).

This set of experiments revealed that IL-12 signaling (1) substantially affects mature oligodendrocytes in the amyloidogenic mouse brain and (2) preferentially acts through an AD-specific upregulation of its ligand IL-12.

### Mature oligodendrocytes are substantially impacted by IL-12 signaling

**Fig. 4:**
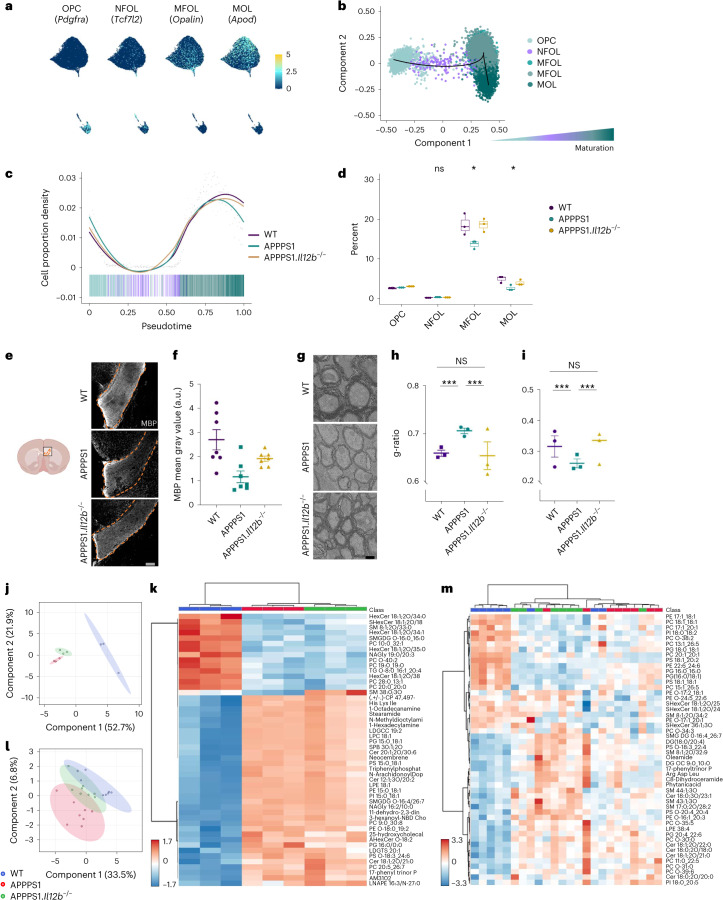
Reduced number of mature oligodendrocytes in the amyloid-carrying hippocampus of AD-like mice exhibit compromised myelin ensheathment. **a**, Feature plots highlighting markers that characterize known oligodendrocyte maturation states. **b**, Pseudotemporal ordering of oligodendrocytes revealed differentiation along the known maturation trajectory from OPC via NFOL to MFOL and MOL. **c**, Cell proportion density along the pseudotime suggests a decrease of more mature oligodendrocytes in the amyloid-carrying APPPS1 mouse hippocampus. **d**, Reduction of oligodendrocytes reaches statistical significance at the stage of MFOL and MOL and is rescued by the absence of IL-12. *n* = 3 per genotype, df = 2, *F* = 2.067 for OPC, *F* = 1.443 for NFOL, *F* = 6.184 for MFOL and *F* = 6.705 for MOL; statistical analysis done by one-way ANOVA with Holm–Bonferroni *P* value adjustment; boxplots show middle, median; lower hinge, 25% quantile; upper hinge, 75% quantile; upper/lower whisker, largest/smallest observation less/greater than or equal to upper/lower hinge ± 1.5× IQR. **e**, Representative immunohistochemical MBP staining of corpus callosum from 250-day-old WT, APPPS1 and APPPS1.*Il12b*^−/−^ mouse brains. **f**, Analysis of MBP mean gray value, normalized by DAPI mean gray value. One-way ANOVA with Tukey’s multiple comparison test, df = 2, *F* = 7.185, **P = 0.0051. Each symbol represents one mouse. Bars represent mean ± s.e.m. **g**, Representative ultrastructural images depicting the hippocampal alveus of 250-day-old WT, APPPS1 and APPPS1.*Il12b*^−/−^ mice. Scale bar, 2 µm. **h**, Analysis of g-ratio depicting the proportion of the inner axonal diameter to the total outer myelin, Kruskal–Wallis chi-squared = 126.83, df = 2, *P* < 2.2 × 10^−16^. **i**, Myelin sheath thickness of *n* = 3 mice per genotype, Kruskal–Wallis chi-squared = 23.244, df = 2, *P* = 8.966 × 10^−6^. Electron microscopy images were analyzed by Kruskal–Wallis rank-sum test with Bonferroni correction for multiple testing. Bars represent mean ± s.e.m. **j**, PLS-DA plot of lipidomics data of 120-day-old WT, APPPS1 and APPPS1.*Il12b*^−/−^ white matter. **k**, Heatmap of lipidomics data of 120-day-old WT, APPPS1 and APPPS1.*Il12b*^−/−^ white matter. **l**, PLS-DA plot of lipidomics data of 250-day-old WT, APPPS1 and APPPS1.*Il12b*^−/−^ white matter. **m**, Heatmap of lipidomics data of 250-day-old WT, APPPS1 and APPPS1.*Il12b*^−/−^ white matter. df, degrees of freedom; IQR, interquartile range; NS, not significant.

**Extended Data Fig. 4:**
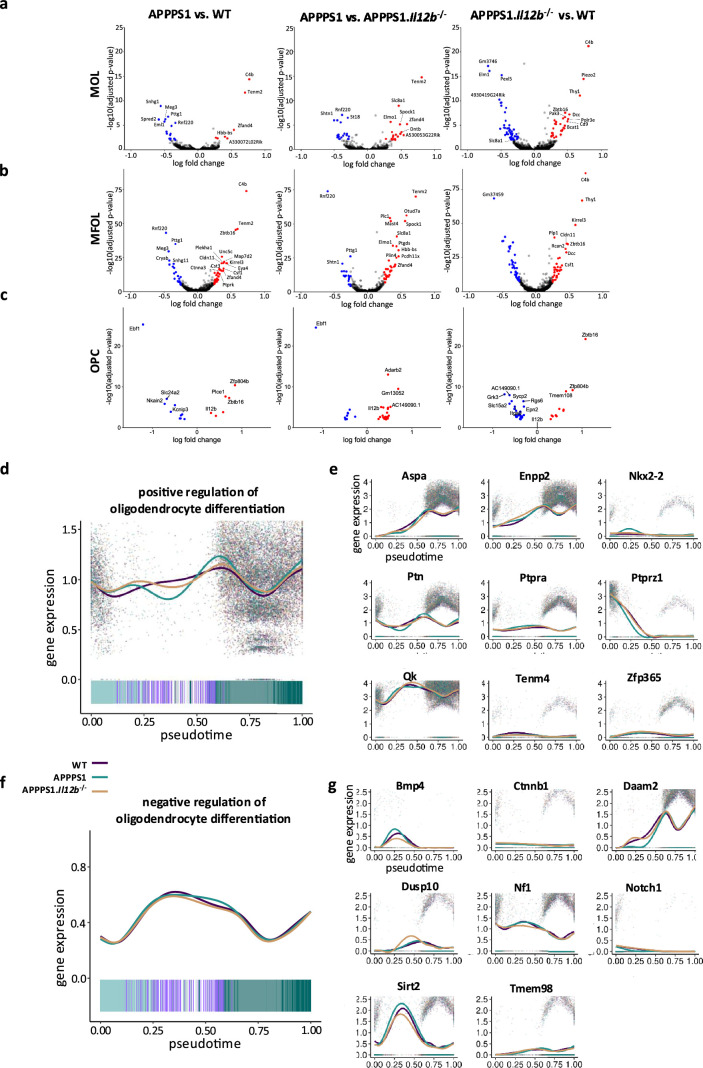
Differential gene expression of oligodendrocyte developmental states show unchanged OPC to oligodendrocyte maturation in amyloid-carrying APPPS1 mice. **a,b,c**. Volcano plots showing differentially regulated genes across all genotypes in MOL (**a**), MFOL (**b**) and OPC (**c**). Pseudotime analysis for genes involved in positive regulation (GO:0048714) of oligodendrocyte maturation (**d-e**) and negative regulation (GO:0048715) of oligodendrocyte maturation (**f-g**) show no strong difference across genotypes.

**Extended Data Fig. 5:**
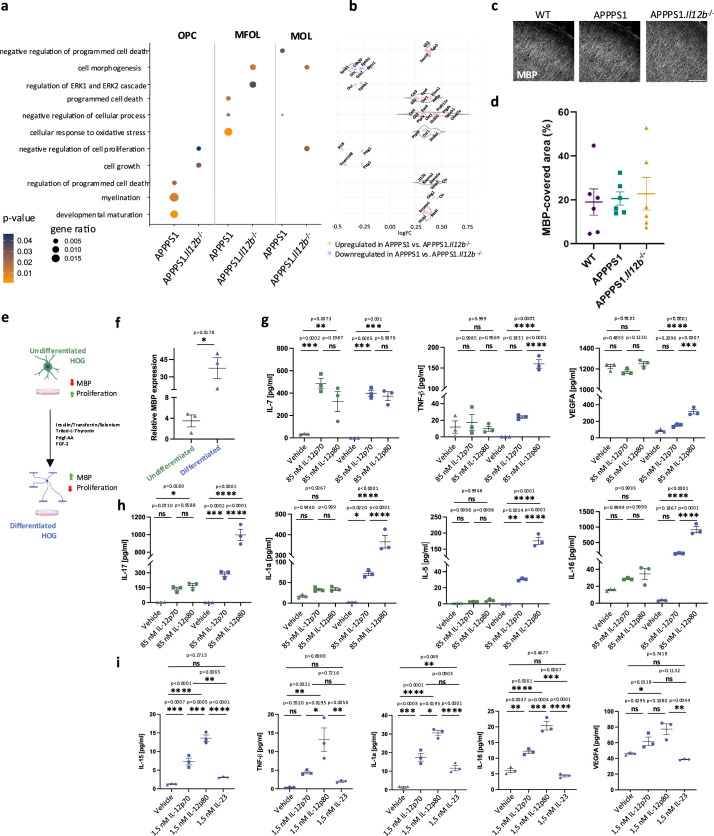
IL-12-dependent status of murine oligodendrocytes and of matured human-oligodendrocyte-like cells. **a**. Gene Ontology (GO) of differentially expressed transcripts of different types of oligodendrocytes derived from APPPS1 vs. APPPS1.*Il12b*^*−/−*^ mice. Dot size illustrates gene ratio, color denotes *P* value. One-way ANOVA with Holm Bonferroni *P* value adjustment. **b**. Violin plot depicting log_2_-fold changes in expression of distinct genes of the analysis shown in (**a**). Red dots indicate genes with positive log_2_-fold changes, blue dots those with negative log_2_-fold changes. **c**. MBP-immunoreactivity in the cortex of APPPS1 and APPPS1.*Il12b*^*−/−*^ versus WT control. Scale bar: 100µm. **d**. Quantification of MBP-covered area, *n* = 6 biological replicates per genotype, one-way ANOVA showed no significant differences. **e**. Cartoon illustrating human oligodendroglioma (HOG) cell maturation. **f**. qPCR of differentiated HOG cell line exemplifying oligodendrocyte-like cells versus non-differentiated HOG cells show a maturation-dependent increase in MBP expression, n = 3 technical replicates, Student’s *t*-test, *P* = 0.0178. **g**. Statistical analysis using two-tailed unpaired students *t*-test, *t* = 3.885, df = 4, *n* = 3 technical replicates. **h**. Maturation/differentiation-dependent increase in the expression of various pro-inflammatory cytokines in the supernatant of HOG upon stimulation with 85 nM IL-12p70, 85 nM IL-12p80 or vehicle only. Undifferentiated HOG cells are shown in green squares, differentiated HOG cells are in blue squares. All df = 17. F = 19.70 for IL-7, F = 85.55 for TNF-ß, F = 876.8 for VEGFA, F = 155.2 for IL-17, F = 117.4 for IL-1a, F = 232.2 for IL-16, *n* = 3 technical replicates. **i** Even doses as low as 1.5 nM IL-12p70 or 1.5 nM IL-12p80 suffice to induce cytokine expression in differentiated HOG cells, while 1.5 nM IL-23 has almost no effect. All df = 11, F = 73.8 for IL-15, F = 12.7 for TNF-ß, F = 67.3 for IL-1a, F = 79.2 for IL-16, F = 10.5 for VEGFA. Analyzed by one-way ANOVA with Tukey’s multiple-comparisons test. Each dot represents one technical replicate (*n* = 3). Bars represent mean ± s.e.m.

By investigating the most highly variable genes in the oligodendrocyte lineage and all expressed transcription factors in our snRNA-seq dataset, we observed a differential trajectory of oligodendrocyte clusters resembling various stages of differentiation or maturation (Fig. 4a,b). A detailed analysis revealed that preferentially mature oligodendrocytes, namely myelin-forming oligodendrocytes (MFOLs) and mature oligodendrocytes (MOLs), accounted for the strong reduction of these cell populations in APPPS1 mice (Fig. 4c,d). To assess whether the reduced number of mature oligodendrocytes might be the result of a dysregulation in OPC maturation in APPPS1 mice, we investigated the genetic profile of the various maturation states of oligodendrocytes (Extended Data Fig. 4a–c). Pseudotime analyses of genes involved in regulating oligodendrocyte differentiation showed no difference between APPPS1 and APPPS1.*Il12b*^−/−^ mice across all oligodendrocyte maturation states (Extended Data Fig. 4d–g). However, Gene Ontology (GO) analysis in OPCs revealed enriched genes involved in myelination (*Clu* and *Olig2*) and developmental differentiation (*Plcb1*, *Kcnma1* and *Sez6l*) in APPPS1 versus APPPS1.*Il12b*^−/−^ mice, indicating compensatory processes in OPCs directed at replacing dysfunctional and/or lost mature oligodendrocytes (Extended Data Fig. 5a). Given that the concept of OPC dynamics, balancing oligodendrocyte cell survival and cell death primarily is known from CNS development, it may well apply and re-appear in the context of CNS diseases affecting myelin and/or mature oligodendrocytes. In MFOLs of APPPS1 mice, genes involved in cellular responses to oxidative stress (*Slc8a1*, *Ptprk* and *Oxr1*) and in programmed cell death (*Elmo1*, *Oxr1*, *Hif3a*, *Gli2*, *Eya4* and *Cst3*) were upregulated when compared to MFOLs derived from APPPS1.*Il12b*^−/−^ mice. Similarly, MOLs of APPPS1 mice showed an upregulation of genes inhibiting programmed cell death (*Sox10*, *Gli2* and *Sgk3*) compared to their counterparts derived from APPPS1.*Il12b*^−/−^ mice (Extended Data Fig. 5b), indicating that IL-12 signaling is capable of regulating oligodendrocyte homeostasis by mediating the balance between cell death and survival.

To assess oligodendroglial myelin sheath-forming capacity in relation to AD pathology and IL-12 signaling, we analyzed myelin integrity by means of immunohistochemistry and electron microscopy. Quantification of myelin basic protein (MBP) as an essential product of MFOLs revealed reduced MBP immunoreactivity in APPPS1 versus WT mice in the corpus callosum. In contrast, levels were unaltered in APPPS1.*Il12b*^−/−^ versus WT mice (Fig. 4e,f). In the somatosensory cortex, MBP coverage was similar across all genotypes, most likely due to the relatively small amount of myelination in this brain area (Extended Data Fig. 5c,d). Using electron microscopy, we analyzed the ultrastructure of myelin in the hippocampus of APPPS1 mice lacking or harboring IL-12 signaling. In line with our previous findings, the proportion of the inner axonal diameter to the total outer myelin (depicted by the g-ratio) was higher in APPPS1 mice (indicating thinner myelin sheaths) compared to WT mice, whereas APPPS1.*Il12b*^−/−^ mouse myelin appeared similar to WT myelin (Fig. 4g–i). The rather high g-ratio observed across all genotypes is likely due to the old age of the animals investigated, as aging itself leads to myelin swelling, fragmentation and delamination^17–19^.

Moreover, lipid profiles generated by untargeted lipidomics of myelin-enriched corpus callosum tissue were clearly distinct in APPPS1 mice compared to WT mice at 120 days and 250 days of age, reflecting the observed myelin pathology. This AD-specific phenotype was partially rescued in 250-day-old APPPS1.*Il12b*^−/−^ mice, indicating that the AD-specific alterations of the CNS lipidome are partially restored upon interference with IL-12 signaling (Fig. 4j–m).

**Fig. 5:**
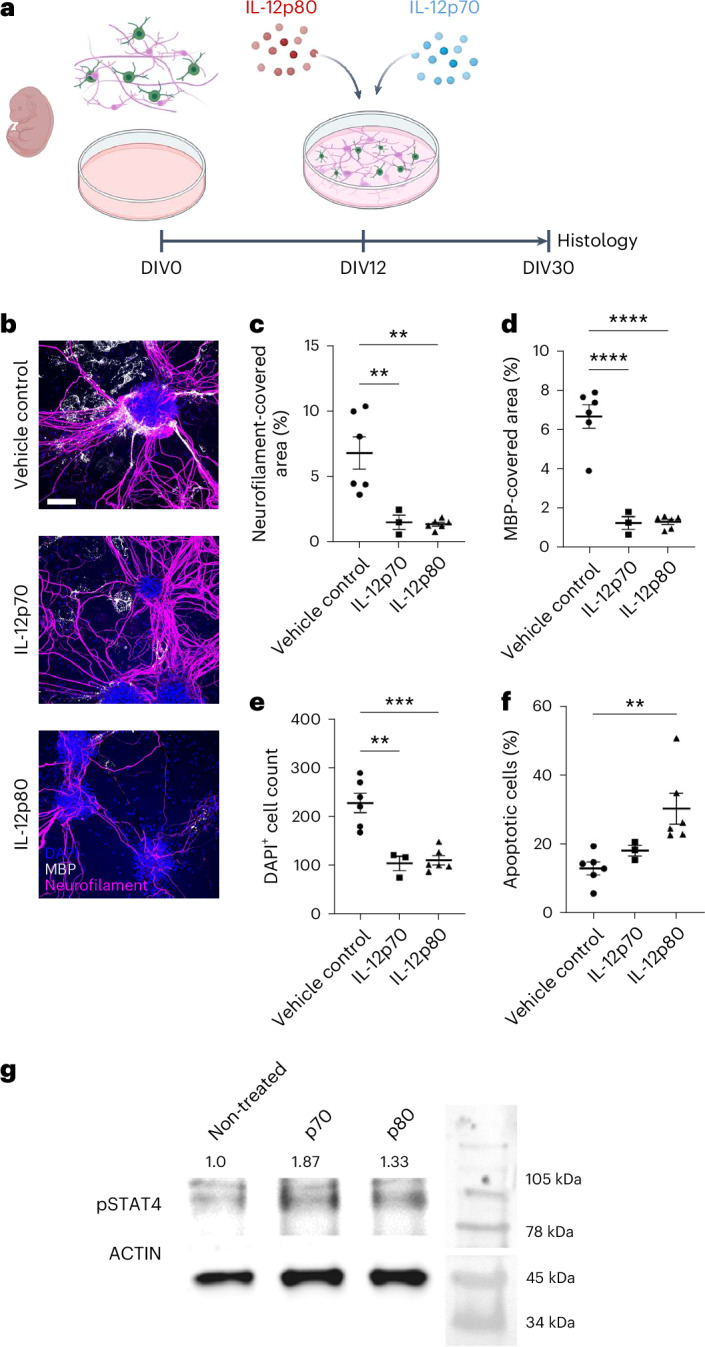
IL-12p70 or IL-12p80 stimulation of murine embryonic primary myelinating culture results in reduced neurofilament and myelination. **a**, Cell culture from E13 murine spinal cords treated with either IL-12p70 or IL-12p80 from DIV12 to DIV30. **b**, Immunocytochemistry from DIV30: blue, DAPI; white, MBP; pink, neurofilament. Scale bar, 100 µm. **c**, Quantification of neurofilament-covered area (%), biological replicates of vehicle-treated and IL-12p80-treated (*n* = 6) and IL-12p70-treated (*n* = 3) cell cultures, one-way ANOVA for vehicle control (mean = 6.780 ± s.e.m.), IL-12p70 (mean = 1.513 ± s.e.m.) and IL-12p80 (mean = 1.368 ± s.e.m.), df = 2, *F* = 13.09, *P* = 0.0010. **d**, Quantification of MBP-covered area (%), biological replicates of vehicle-treated and IL-12p80-treated (*n* = 6) and IL-12p70-treated (*n* = 3) cell cultures, one-way ANOVA for vehicle control (mean = 6.682 ± s.e.m.), IL-12p70 (mean = 1.237 ± s.e.m.) and IL-12p80 (mean = 1.295 ± s.e.m.), df = 2, *F* = 52.70, *P* < 0.0001. **e**, DAPI^+^ cell count, biological replicates of vehicle-treated and IL-12p80-treated (*n* = 6) and IL-12p70-treated (*n* = 3) cell cultures, one-way ANOVA for vehicle control (mean = 227.0 ± s.e.m.), IL-12p70 (mean = 104.0 ± s.e.m.) and IL-12p80 (mean = 110.2 ± s.e.m.), df = 2, *F* = 19.80, *P* = 0.0002. **f**, Quantification of apoptotic cells (%), biological replicates of vehicle-treated and IL-12p80-treated (*n* = 6) and IL-12p70-treated (*n* = 3) cell cultures, Kruskal–Wallis test for vehicle control (mean = 3.832 ± s.e.m.), IL-12p70 (mean = 7.333 ± s.e.m.) and IL-12p80 (mean = 12.50 ± s.e.m.), Kruskal–Wallis statistic = 11.35, *P* < 0.0001. **g**, Actin and pSTAT4 western blot analysis of IL-12p70-treated, IL-12p80-treated and non-treated primary oligodendrocytes; non-treated: 1.0 (left), IL-12p70-stimulated: 1.87 (center) and IL-12p80: 1.33 (right); pSTAT4 signal normalized to actin signal. df, degrees of freedom. Source data

To confirm the direct negative impact of IL-12p70 and IL-12p80 signaling on oligodendrocyte and neuronal homeostasis, we administered IL-12p70 and IL-12p80 to primary murine co-cultures consisting of neurons, microglia, astrocytes and oligodendrocytes; the latter typically form compact myelin sheaths around neurofilament-positive axons^20^ (Fig. 5a). This treatment reduced the density of MBP and neurofilament compared to vehicle-control-stimulated cultures (Fig. 5b–d), impacted overall cell survival as measured by cell numbers and enhanced cleaved caspase-3 immunoreactivity (Fig. 5e,f) and was mediated by phosphorylated STAT4 (Fig. 5g).

To test whether human oligodendrocytes have, in principle, the capacity to react to IL-12 on a functional level, we differentiated human oligodendrocyte-like cells derived from the oligodendroglioma cell line SCC163 cells in vitro^21^ (Extended Data Fig. 5e,f) and treated these cells with IL-12p70, IL-12p80 or IL-23. Upon maturation, human oligodendrocyte-like cells released an array of pro-inflammatory factors, such as TNF-β, IL-1a and IL-17, when exposed to IL-12, of which IL-12p80 induced the strongest response (Extended Data Fig. 5g,h), whereas there was almost no response to IL-23 (Extended Data Fig. 5i).

In conclusion, these data show that myelination is substantially impaired in the amyloid-rich mouse brain in an IL-12-dependent fashion—a phenotype that can be rescued in vivo through the deletion of IL-12 signaling—whereas mouse oligodendrocytes and human and oligodendrocyte-like cells react profoundly to IL-12 treatment in vitro.

### IL-12 signaling affects neuronal homeostasis in AD-like mice

**Fig. 6:**
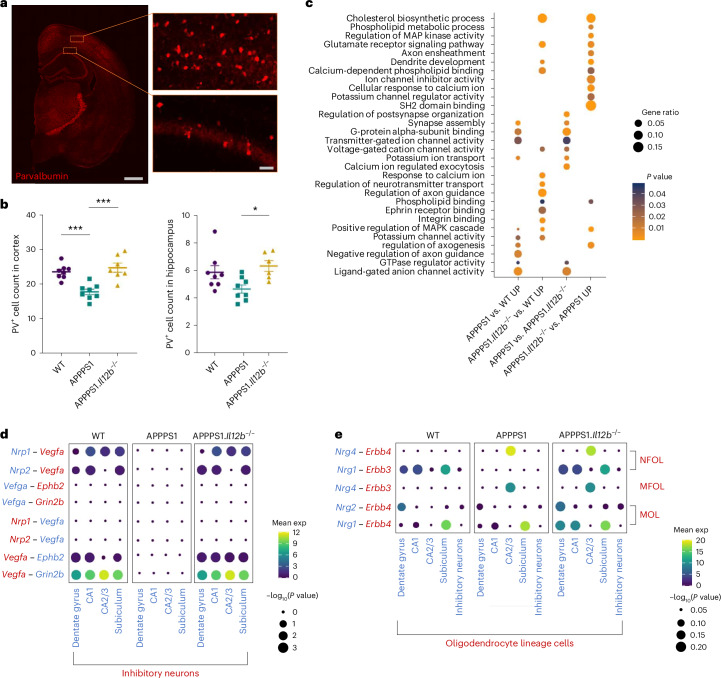
IL-12 signaling leads to transcriptional changes in mouse hippocampal and cortical neurons. **a**, Representative images of PV^+^ interneurons in the cortex and hippocampus of APPPS1.*Il12b*^−/−^ mice. **b**, Quantification of PV^+^ interneurons comparing WT (cortex mean = 23.49 ± s.e.m.; CA1 mean = 5.868 ± s.e.m.), APPPS1 (cortex mean = 17.67 ± s.e.m.; CA1 mean = 4.653 ± s.e.m.) and APPPS1.*Il12b*^−/−^ mice (cortex mean = 24.66 ± s.e.m.; CA1 mean = 6.324 ± s.e.m.) (*n* = 8 per genotype), one-way ANOVA, df = 2, *F* = 4.620, *P* = 0.232 for CA1 and one-way ANOVA, df = 2, *F* = 15.68, *P* < 0.0001 for cortex. **c**, GO analysis of genes upregulated in subiculum comparing APPPS1 versus WT mice, APPPS1.*Il12b*^−/−^ versus WT mice, APPPS1 versus APPPS1.*Il12b*^−/−^ mice and APPPS1.*Il12b*^−/−^ versus APPPS1 mice. **d**,**e**, Using CellPhoneDB, dot plot showing the predicted receptor–ligand interactions between neuronal cell types (**d**) and oligodendrocytes (**e**) in WT, APPPS1 and APPPS1.*Il12b*^−/−^ mice. *P* values are indicated by the circle size, and means of the average expression level are color coded. df, degrees of freedom; exp, expression.

We previously showed that pharmacological inhibition or genetic deletion of IL-12/IL-23 signaling reversed cognitive deficits in aged APPPS1 mice^7^, suggesting that—based on the herein-presented identification that IL-12, but not IL-23, is key in AD-associated IL-12/IL-23 signaling—neuronal homeostasis is affected by IL-12 signaling. Approximately 10–15% of neurons in the hippocampus are GABAergic inhibitory interneurons^22^, which are known to play a crucial role in determining and regulating cortical circuit function. Cognitive decline in patients with AD, at least in part, can be attributed to neuronal hyperexcitability caused by GABA inhibitory interneuron dysfunction in the hippocampus^23^. Notably, by means of immunohistochemical analyses of brain tissue, we found parvalbumin-positive (PV^+^) interneurons in the hippocampal CA1 region of APPPS1 mice to be decreased when compared to WT littermates (Fig. 6a). Strikingly, this reduction in PV^+^ interneurons was rescued in the hippocampus and the cortex of APPPS1.*Il12b*^−/−^ mice (Fig. 6b).

**Extended Data Fig. 6:**
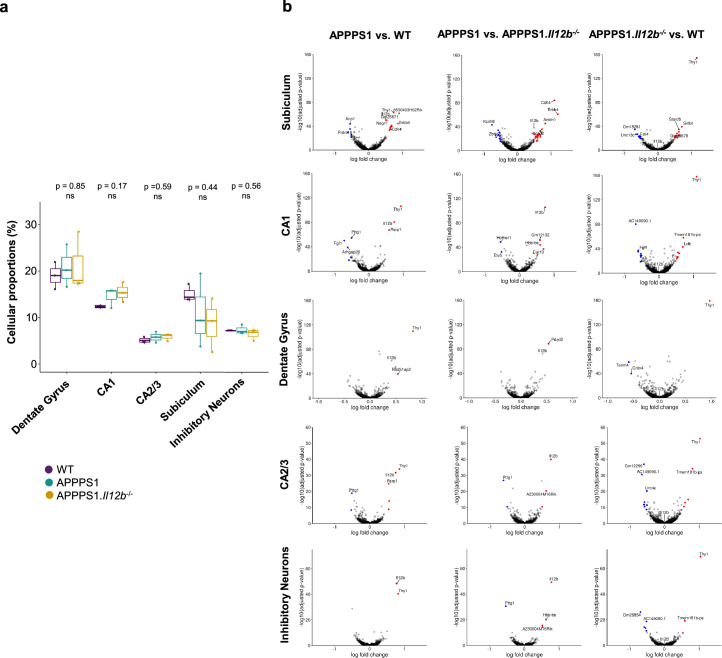
IL-12 signaling leads to transcriptional changes in neurons. **a**, Subicular neurons appeared to be somewhat reduced in APPPS1 and APPPS1.*Il12b*^*−/−*^ mice when compared to WT mice. All df = 2, F = 0.161 for dentate gyrus, F = 2.384 for CA1, F = 0.574 for CA2/CA3, F = 0.935 for subiculum, F = 0.633 for inhibitory neurons, n = 3 biological replicates per genotype. Statistical analysis done by one-way ANOVA with Holm-Bonferroni *P* value adjustment; boxplots show: middle, median; lower hinge, 25% quantile; upper hinge, 75% quantile; upper/lower whisker, largest/smallest observation less/greater than or equal to upper/lower hinge ± 1.5 x IQR. **b**, Volcano plots showing differentially regulated genes across genotypes in subiculum, dentate gyrus, CA1, CA2/3 and inhibitory neurons. ANOVA with Holm-Bonferroni *P* value adjustment. Red and blue dots are determined as statistically significant with p ≤ 0.01 and log2 fold-change ± > 0.5.

When investigating the effects of IL-12 signaling on neurons at the transcriptome level, we also found a reduced number of neurons within the subiculum in AD-like APPPS1 mice compared to WT littermates (which did not reach significance) as well as alterations in the gene expression of subicular neurons of APPPS1 mice lacking *Il12b* compared to APPPS1 mice with functional IL-12 signaling (Extended Data Fig. 6a,b). Affected genes are involved in pathways impacting hippocampal memory and synaptic plasticity, such as *Erbb4* and *Rarb*^24–26^. In addition to seeing some differential regulation of genes involved in ion homeostasis, we also found an upregulation of genes implicated in dendrite development (*Dab1*, *Fat3*, *Fezf2*, *Fmn1*, *Hecw2*, *Klhl1*, *Map1b*, *Nedd3l*, *Sez6* and *Ss181*) in APPPS1.*Il12b*^−/−^ mice compared to APPPS1 mice, suggesting IL-12-dependent enhanced compensatory efforts aimed at regenerating neuronal homeostasis in the AD microenvironment (Fig. 6c).

To gain insights into the cell–cell communication between different neuronal subtypes, we applied CellPhoneDB, a publicly available repository of distinct receptors, ligands and their interactions, to provide correlative relations of co-expressed receptor–ligand pairs derived from single-cell RNA-seq data^27^. We observed a region-specific IL-12-dependent alteration in the receptor–ligand pairing of neuropilin (*Nrp*) 1, *Nrp2* and vascular endothelial growth factor A (*Vegfa*) as well as *Ephb2* and *Grin2b* in excitatory and inhibitory neurons in APPPS1 mice, in subiculum, dentate gyrus and CA2/CA3 (Fig. 6d). *Nrp1* and *Nrp2* serve as co-receptors for VEGF receptors and support neuronal guidance as well as dendritic growth and branching in the adult brain^28,29^. Of note, CellPhoneDB-based analyses revealed a reduced *Nrg1–Erbb3* interaction in newly formed oligodendrocytes (NFOLs) in the dentate gyrus, CA1 and in the subiculum of APPPS1 compared to WT mice and was rescued in APPPS1.*Il12b*^−/−^ mice, reaching WT levels (Fig. 6e). This signaling affects oligodendrocyte survival in vitro^30^ but, so far, has been claimed to be involved only in normal myelination in vivo in the peripheral nervous system by gain of function^31^. Moreover, *Nrg2–Erbb4* was reduced in dentate gyrus–derived MOLs of APPPS1 mice and reverted to WT levels in APPPS1.*Il12b*^−/−^ mice lacking functional *Il12b* signaling. These data indicate that IL-12 signaling-dependent perturbations in the transcriptional profile of neurons (ultimately leading to functional dysregulation) might be mediated either through binding of IL-12 to its receptor directly on the neuronal cell surface or through IL-12 affecting oligodendrocytes and leading to an alteration in their trophic support of neurons^32^ or a combination thereof.

### Altered microglial function in AD-like APPPS1.*Il12b*^−*/*−^ mice

**Fig. 7:**
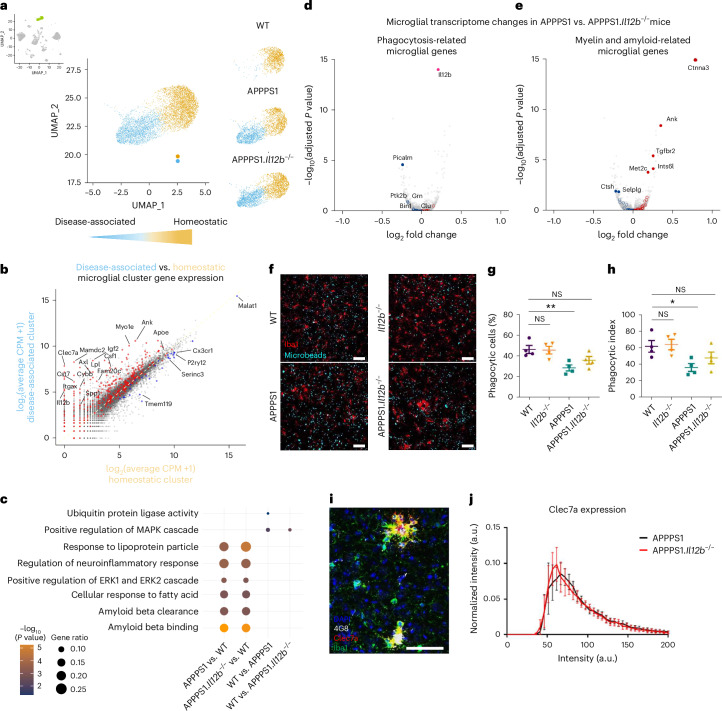
Microglia in APPPS1 and APPPS1.*Il12b*^−/−^ mice share gene signatures associated with enhanced microgliosis but exhibit distinct phagocytotic phenotypes. **a**, Distinct homeostatic (yellow) and disease-associated (blue) microglia clusters were found in the combined snRNA-seq dataset with the disease-associated clusters present only in APPPS1 and APPPS1.*Il12b*^−/−^ mice. **b**, Scatterplot comparing the gene expression in the disease-associated clusters versus the homeostatic clusters. **c**, GO analysis of differentially upregulated genes per indicated genotypes. The dot size illustrates gene ratio, and the color denotes *P* value. Violin plot showing log_2_FC of certain specific genes to the corresponding GO term. Fisher’s exact test and the GO algorithm ‘elim’. **d**,**e**, Volcano plots showing differentially expressed genes in microglia of APPPS1 mice compared to APPPS1.*Il12b*^−/−^ mice (downregulated: blue; upregulated: red) known to be involved in phagocytosis of microglia (**d**) or to be myelin related or amyloid related (**e**). Adjusted *P* value by Benjamini–Hochberg. A cluster of selected AD risk genes involved in phagocytosis in ex vivo human microglia and human brain lysates^68,69^ served as reference for assessing phagocytosis-related microglial transcriptome changes, which, upon conversion into their mouse orthologs, resulted in 27 genes comprising *Bin1*, *Ptk2b*, *Trem2*, *Zyx*, *Apbb3*, *Clu*, *Rin3*, *Cd33*, *Ms4a4a*, *Cr1l*, *Grn*, *Apoe*, *Picalm*, *Cd2ap*, *Plcg2*, *Sorl1*, *Fermt2*, *Ap4e1*, *Zkscan1*, *Abca7*, *Siglech*, *Trp53inp1*, *Abi3*, *Rabep1*, *Cass4*, *Ap4m1* and *Sppl2a*. Myelin-related or amyloid-related transcriptome changes in microglia (right) were defined by referencing the gene list described by Depp et al.^32^ (Supplementary Table 1, tab 6) depicting differentially expressed genes of DAM derived from 6-month-old mice with amyloid pathology and/or mutant myelin, followed filtering by logFC > 0.25 and FDR < 0.01. Genes that were altered significantly are shown as filled circles (FDR < 0.05); open circles indicate differences that did not reach statistical significance. *Il12b* served as internal control. **f**–**h**, Phagocytic activity of microglia in adult acute brain slices of WT and APPPS1 mice with and without IL-12 signaling. Organotypic brain slices prepared from 90-day-old WT (*Il12b*^*+/+*^*)*, *Il12b*^−/−^, *APPPS1* and APPPS1.*Il12b*^−/−^ mice were incubated with fluorescent microbeads to analyze phagocytic microglia. Representative views from 15-μm confocal z-stacks showing uptake of fluorescent microbeads (in green) by microglia (labeled with Iba1, red) in brain slices of mice with the indicated genotypes (**f**). Percentage of phagocytic microglia with engulfed microbeads (*P* = 0.0104) (**g**) and phagocytic index (*P* = 0.0314) (**h**). For the calculation of the phagocytic index, phagocytic cells were grouped according to the number of ingested microbeads, with 1–3 microbeads = grade 1; 4–6 microbeads = grade 2; 7–10 microbeads = grade 3; and more than 10 microbeads = grade 4. Each grade (1–4) was multiplied with the respective percentage of phagocytic microglia to calculate the phagocytic index. Scale bar, 50 μm. *n* = 4 mice per group (mean ± s.e.m., one-way ANOVA with Dunnett’s post hoc test with WT as control group). **i**, Representative immunohistochemical image of Clec7a, Iba1 and 4G8 staining in APPPS1.*Il12b*^−/−^ mouse brain cortical tissue. Scale bar, 100 µm. **j**, Clec7a staining intensity within plaque‐associated Iba1^+^ microglia in WT and APPPS1.*Il12b*^−/−^ mice (*n* = 6). Mean ± s.e.m., statistical analysis: two‐tailed unpaired *t*‐test with Bonferroni correction for each single bin, *P* = NS. NS, not significant.

**Extended Data Fig. 7:**
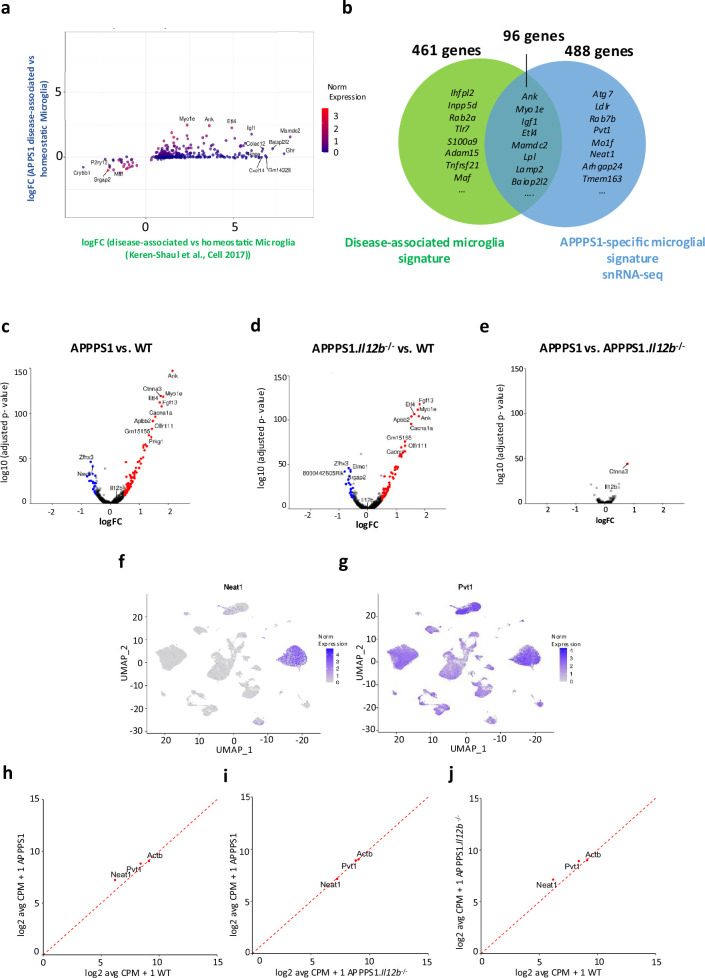
Comparing publicly available inflammatory protein and non-protein coding gene signatures of 5xFAD mice to those of APPPS1 mice. **a-b**. Comparing published gene signatures of disease-associated microglia (DAM) and homeostatic microglia (green, b) from 5xFAD AD-like mice to respective microglia signatures from APPPS1 AD-like mice generated within this study reveals (blue, b) a substantial overlap. **c-e** Volcano plots comparing gene expression from all microglial cells in APPPS1 versus WT mice (**c**), APPPS1.*Il12b*^*−/−*^ vs. WT mice (**d**) and APPPS1 vs APPPS1.*Il12b*^−/−^ mice (**e**). **f-g**, UMAP feature plots showing non-coding transcripts for *Neat1* (**f**) and *Pvt1* (**g**). **h-j**, Volcano plot comparing APPPS1 vs. WT mice (**h**), APPPS1 vs. APPPS1.*Il12b*^*−/−*^ mice (**i**) and APPPS1.*Il12b*^*−/−*^ vs. WT mice (**j**) illustrating that the non-coding lncRNA *Neat1* and *Pvt1* were upregulated in both AD-related genotypes.

Because microglia have a strong impact on neuroinflammation and AD pathology, we assessed transcriptional changes in microglia from APPPS1 mice with and without genetic ablation of *Il12b*. We observed two microglia clusters (Fig. 7a), one of which was prominent in both APPPS1 and APPPS1.*Il12b*^−/−^ mice—that is, AD specific but virtually absent in age-matched WT mouse brains. Differential gene expression analyses between these two microglia clusters showed upregulation in IL-12-competent and IL-12-deficient APPPS1 mice of few *Trem2*-independent (*Apoe*) and many *Trem2*-dependent (*Ank*, *Csf1*, *Clec7a*, *Axl*, *Spp1*, *Itgax* and *Igf1*) genes^33^, including lipid metabolism and phagocytic pathways (*Lpl*, *Cst7* and *Cd9*) as well as the downregulation of homeostatic microglia genes (*P2ry12*, *Cx3cr1* and *Tmem119*) (Fig. 7b). This gene expression signature showed strong similarities to the previously described signature from disease-associated microglia (DAM), which is linked to an altered microglial activation state in another AD mouse model, namely 5×FAD^33^, including a modified functional status and secretion of neuroinflammatory mediators. Apart from an overlap with many previously described DAM genes (Extended Data Fig. 7a and Supplementary Data Table 2) and in light of the fact that the DAM signature typically is assigned to the cytoplasm of microglia and, thus, not entirely covered by our approach of applying snRNA-seq^34^, we also identified significantly differentially regulated genes (*Atg7*, *Ldlr*, *Rab7b*, *Pvt1*, *Mo1f*, *Neat1*, *Arhgap24* and *Tmem163*) in microglia of APPPS1 mice, which were not previously described as part of the DAM signature (Extended Data Fig. 7b and Supplementary Data Table 3).

Surprisingly, the inflammatory microglial gene signature of APPPS1 mice was largely unaffected by IL-12/IL-23 signaling, resulting in a similar differential gene regulation in microglia from both APPPS1 and APPPS1.*Il12b*^−/−^ mice when compared to WT mice (Extended Data Fig. 7c–e). GO analysis showed that genes involved in ubiquitin protein ligase activity (*Mycbp2*, *Asb2*, *Rnf216*, *Rnf130* and *Znrf1*), thought to be linked to regulating neuroinflammation in AD^35^, were found to be slightly but significantly upregulated in WT versus APPPS1 mice but not significantly in APPPS1.*Il12b*^−/−^ mice (Fig. 7c). Of note, we identified differentially expressed microglial genes known to be involved in phagocytosis (for example, *Picalm*) (Fig. 7d) or to be myelin related or amyloid related (Fig. 7e) when comparing microglia of APPPS1 mice to those of APPPS1.*Il12b*^−/−^ mice. To validate the observed alterations in microglial genes affecting phagocytosis of Aβ on a functional level, we assessed the activity of microglia in phagocytosing fluorescent beads in situ using organotypic brain slice cultures derived from WT and APPPS1 mice with or without functional IL-12 signaling (Fig. 7f). In accordance with the changes on the transcriptional level, the phagocytic activity of microglia from APPPS1 mice—both at the level of phagocytic cells and of the phagocytic index—was significantly reduced compared to WT microglia. Notably, microglia derived from APPPS1 mice lacking IL-12 signaling displayed a rescued phagocytic activity, where IL-12-deficient microglia were as efficient in eating up fluorescent beads as WT microglia (Fig. 7g,h), despite the fact that Clec7a (Dectin-1) intensities were not altered in Iba1^+^ microglia adjacent to 4G8^+^ Aβ plaques in APPPS1 versus APPPS1.*Il12b*^−/−^ mice (Fig. 7i,j).

In addition to protein-coding RNAs, non-coding RNAs are part of the microglial immune response^36,37^. Because the non-coding linear *Pvt1* and *circPvt1* as well as *Neat1* are capable of regulating the immune response^38,39^, we assessed their expression in APPPS1 mice with or without functional p40 signaling. *Pvt1* and *Neat1* were equally and indistinguishably upregulated in APPPS1 and APPPS1.*Il12b*^−/−^ mice when compared to WT mice (Extended Data Fig. 7f–j), corroborating our finding that the disease-associated coding and non-coding gene signature of microglia in Aβ-bearing APPPS1 mice is independent of IL-12 in the hippocampus.

## Discussion

Our data unequivocally provide evidence that the IL-12/IL-23p40-mediated amelioration of AD pathology previously described by us^7,8^ is mediated solely through IL-12 signaling, whereas IL-23 plays no role. Furthermore, our chosen approach of deleting IL-12 and IL-23 signaling in neuroectodermal cells of AD-like APPPS1 mice allows us to conclude that the crosstalk of AD-specific microglia-produced IL-12p40 (refs. ^7,8^) acts through neuronal and oligodendroglial IL-12 receptors, as oligodendrocytes and neurons harbor the respective transcripts, namely *Il12rb1* and *Il12rb2*. These transcripts were found to be equally present in AD-like and WT mice, with more cells expressing *Il12rb2*, whereas *Il12rb1* and *Il23r* expression—the latter of potential relevance for settings other than AD, where IL-23R signaling in the CNS may play a role—was restricted to fewer cells. Given that each of the two IL-12 receptor subunits can occur as dimers/oligomers differing only in their affinity to bind IL-12 (refs. ^4–6^), imbalanced subunit expression will not necessarily be rate-limiting in executing IL-12 actions. In contrast to the constitutive expression of IL-12 receptors, the expression of *Il12b* encoding for IL-12 ligand mainly by microglia was shown to occur AD-specifically in mice^7^. As the genetic repertoire for IL-12 signaling, namely *IL12RB1* and *IL12RB2* transcripts, is also present in neurons and oligodendrocytes in postmortem human primary motor cortex according to the Allen Brain Map, and because we found IL-12 receptor transcripts, but not those for IL-23 receptor, in postmortem hippocampi of human AD and control patients, the CNS IL-12/IL-23 signaling repertoire seems to be similar in mice and humans. In view of significantly increased IL-12p70 protein levels in AD brains compared to non-demented control cases, where higher Braak stages—indicative of a stronger cognitive decline—correlated with higher IL-12p70 protein expression, and given the profound response of human oligodendrocyte-like cells to IL-12 stimulation in vitro—in contrast to IL-23, which showed almost no effects—IL-12 appears to be an attractive and obvious druggable target in AD. Noteworthy, this contrasts with peripheral autoimmune diseases mediated through IL-12/IL-23 signaling, such as psoriasis or Crohn’s disease, where IL-23 has been identified as the main driver^40^. In line with our data, Andreadou et al.^41^ demonstrated that oligodendrocytes and neurons are the IL-12 receptor-bearing cell types in brain tissue of patients with multiple sclerosis (MS) and of experimental autoimmune encephalitis (EAE) mice, an inducible animal model of MS. However, in EAE, where pathology is mainly triggered by CNS-infiltrating peripheral immune cells, IL-12 has been shown to have a neuroprotective effect^41^, indicating that IL-12 appears to be yet another molecule with dual functions in inflammation versus degeneration of the CNS, as also shown for TREM2 (refs. ^42,43^) or granulocyte-macrophage colony-stimulating factor (GM-CSF)^44,45^. This can be explained by differences in the inflammatory context in the respective model, namely the amount, the cellular sources and duration of IL-12 action (rather short and high through monocyte-derived cells in EAE versus chronic and low in glial cells in AD-like mice).

### The oligodendroglial site of AD-related IL-12 signaling

How to interpret the herein-described IL-12-dependent reduction of mature oligodendrocytes in Aβ-overexpressing APPPS1 mice? Although there is general agreement on the occurrence of white matter changes and myelin pathology in human patients with AD based on imaging and postmortem data^46^, animal studies using AD-like mice are inconclusive in this respect—possibly due to the fact that white and gray matter of the CNS differ in oligodendrocyte densities. While in vivo studies suggest that oligodendrocytes may be affected by the Aβ burden, resulting in a decrease in MBP^47–49^, other studies report that Aβ exposure to oligodendrocytes in vitro induces a more mature phenotype, as evidenced by an increase in oligodendrocyte branching and MBP production^50^. By comparing the overall oligodendrocyte numbers in AD-like mice and patients with AD, it has been suggested that the change in numbers reflects their dynamic alterations depending on the disease state^51^. Although previous studies reported an increase in Olig2^+^ cells in the cortex of Aβ-overexpressing APPPS1 (ref. ^51^) and 5×FAD mice^52^, we found a substantial reduction of mature CC1^+^/Olig2^+^ oligodendrocytes in the hippocampus, cortex and corpus callosum of amyloid-harboring and neuroinflamed APPPS1 mice, which is entirely rescued to WT levels upon deleting IL-12. On a transcriptional level, we found no gross alterations in genes involved in regulating oligodendrocyte differentiation and maturation, whereas mature oligodendrocytes—presumably based on their IL-12/IL-23 signaling repertoire—were affected by the AD-specific amyloidogenic and inflammatory microenvironment. Similarly, IL-12 affected the myelination capacity of oligodendrocytes: immunohistochemical and ultrastructural analyses revealed a reduction in MBP and myelin sheath thickness in APPPS1 versus WT mice subsequently to the amyloid pathology at 250 days of age, which was restored in APPPS1.*Il12b*^−/−^ mice. Along that line, treatment of co-cultures consisting of oligodendrocytes and neurons with IL-12p70 and IL-12p80 resulted in a decrease in myelination of neurofilament-positive axons as well as an overall decrease in cell numbers, which was accompanied by an increase in cleaved caspase-3, indicating IL-12-mediated cell death. Thus, our data corroborate previous findings on myelin pathology preceding AD pathology: loss of myelin integrity has been shown to be associated with the accumulation of Aβ in imaging studies^53^, in postmortem human brain tissue^54^ and experimentally in several mouse models of AD, namely 3×Tg-AD, Tg2576, APPPS1, 5×FAD and *APP*^*NLGF*^, where myelin abnormalities impair clearance of Aβ^32,55,56^. Myelin perturbation was shown to increase the risk of developing or exacerbating AD—for example, by altering components of the Aβ-generating machinery, including the amyloid precursor protein (APP) and the APP-cleaving enzyme BACE1 (refs. ^32,57^), or by distracting microglia-mediated breakdown of Aβ due to redeployment of these cells to sites of myelin damage^32^. Because IL-12 signaling in oligodendrocytes acts through STAT4/pSTAT4 (ref. ^58^), which can lead to an increase in interferon-γ known to generate myelin and oligodendrocyte loss in aged mice^59^, the co-occurrence and interplay of neuroinflammation and myelin defects may exacerbate AD pathology by creating a ‘vicious circle’.

### The neuronal site of AD-related IL-12 signaling

In addition to substantially affecting oligodendrocytes, IL-12/IL-23 signaling also altered the gene signatures of subicular neurons of the hippocampus—a brain region of high relevance for cognitive functions. Atrophy in the subiculum is thought to be the earliest sign of neuronal degeneration in AD^11^ and is connected to memory loss^60^. The subiculum’s role in controlling hippocampal output is substantially influenced by its inherent GABAergic activity: local GABAergic signaling limits the spread of incoming excitation and alters the activity patterns of the subiculum’s principal cells (PCs). Notably, local inhibition impacts intrinsically bursting (IB) neurons, which are thought to be key in the output activity of the subiculum, therefore shaping memory^60^. Studies have reported that neuronal cell death and ultimately cognitive decline in AD can be attributed to hyperexcitable microcircuits due to a loss of GABAergic interneurons. Quantification of PV^+^ interneurons in the hippocampus revealed a hitherto unrecognized reduction of GABAergic interneurons in APPPS1 compared to WT mice, which was restored in IL-12-deficient littermates. These data provide a morphological substrate for the impaired cognitive performance in these mice^7^ and corroborate the IL-12-mediated impact on the neuronal phenotype in these mice.

Although subicular neurons were decreased in APPPS1 mice irrespective of *Il12b*, their transcriptional signature was altered, depending on the expression of IL-12, for genes involved in memory and synaptic plasticity, such as *Erbb4* and *Rarb*, as well as genes regulating dendrite development, such as *Nrp1* and *Nrp2* encoding Neuropilin-1 and Neuropilin-2. As KEGG signaling pathway analysis closely relates NRP1 regulation to the AKT pathway that can get activated by IL-12, and as NRP1 depletion was shown to attenuate phosphorylation of AKT, GSK3β and mTOR proteins^61^, IL-12 may well directly impact NRP1-mediated dendrite development. Another IL-12-mediated mechanism affecting neuronal homeostasis is *Nrg*–*ErbB* signaling, which links oligodendrocyte and neuronal interaction and mediates a plethora of cellular functions in both cell types^62^, including *Nrg1–ErbB*-driven regulation of axonal pathfinding, synaptic behavior and neuronal migration^63,64^.

### Mechanism of action

From a mechanistic viewpoint, the reduction of Aβ burden upon IL-12 deficiency appears to occur by an increase in Aβ clearance, as the APP transgene and genes involved in its processing were not substantially altered in neurons of APPPS1 versus APPPS1*.Il12b*^−/−^ mice, and APP protein expression and its respective processing machinery, including the degradation enzymes, were shown to be unaltered in APPPS1 mice deficient in IL-12 signaling^7^. The fact that microglia from APPPS1 mice lacking IL-12 signaling appear to be less distracted by myelin debris may well explain why they have a greater capacity to successfully clear Aβ than microglia of APPPS1 mice with functional IL-12 signaling. This assumption is based not only on myelin-related and amyloid-related alterations in the transcriptome of microglia and by their increase in expression of phagocytosis-related genes, such as *Picalm*, but also on a functional level, where microglia within organotypic brain slices from APPPS1 mice lacking IL-12 signaling displayed a phagocytic activity alike WT mice, whereas APPPS1 control microglia presented with a substantially reduced capacity to phagocytose. At the same time, the lack of IL-12/IL-23 signaling in AD-like APPPS1 mice did not change the inflammatory state of IL-12/IL-23(p40)-producing microglia substantially, including parts of the DAM APPPS1-specific signatures. One possible explanation is that capturing nuclear RNA by snRNA-seq may exclude cytoplasmic RNA, which is part of the presently defined full DAM signature spectrum^33^. Apart from observing an overlap with the previously described DAM genes, such as *Axl*, *Clec7* and *Cd9*, we also discovered a distinct set of microglia-specific disease-associated genes. As inflammatory transcriptional DAM signatures are thought to cause many changes in microglia, this notable lack of more obvious changes of IL-12/IL-23(p40)-producing microglia apart from those related to their phagocytic capacity may be because paracrine signaling of microglia affects primarily other IL-12/IL-23 recipient-prone CNS-intrinsic cells. This notion is supported by the fact that microglia (including those expressing the activation marker Clec7a) from APPPS1 and APPPS1.*Il12b*^−/−^ mice are similarly distributed around amyloid plaques, suggesting that the described changes in APPPS1.*Il12b*^−/−^ mice are mediated mainly through the above-described IL-12 receptor-triggered oligodendrocyte and/or neuronal phenotype(s). Further evidence for this interpretation comes from the fact that aging-associated myelin dysfunction has recently been shown to result in a stark increase of amyloid deposition around swollen myelin in AD-like mice preferentially in the alveus of the hippocampus but not in the fimbria^32^. The herein-reported lack of changes in microglia of APPPS1.*Il12b*^−/−^ versus APPPS1 mice in light of the pronounced loss of mature oligodendrocytes and impaired myelin integrity may, thus, indicate that the reduction of amyloid upon IL-12 deficiency is rather a result of the inhibition of the AD-specific IL-12-dependent disintegration of myelin (driving enhanced Aβ deposition) than a consequence of an increase in microglial phagocytosis.

### Limitation of the study

Given the pronounced oligodendrocyte phenotype in APPPS1 mice, our study cannot discriminate whether the described neuronal gene alterations as well as the reduction in PV^+^ interneurons are a secondary, indirect effect resulting in oligodendrocyte/myelin alterations or whether the observed neuronal changes are directly IL-12 mediated, which may induce subsequent oligodendrocyte/myelin pathology. The close functional relationship between neurons and oligodendrocytes, for example due to the trophic support of neurons by oligodendrocytes, makes both scenarios, or a mixture thereof, possible. Dissecting the precise sequence of IL-12-mediated alterations will, thus, be a topic of future investigations.

In conclusion, our data are not only instrumental in dissecting the mechanism of IL-12/IL-23-specific immunomodulation of AD by identifying its cellular targets, namely oligodendrocytes and neurons, and by linking myelin changes with the capacity of microglia to phagocytose Aβ; they also highlight the potential of an IL-12/IL-23 targeted immunotherapy in AD. The fact that IL-12, but not IL-23, is the pathogenetically relevant pathway in AD-related IL-12/IL-23 signaling—of note and in contrast to other IL-12/IL-23-mediated (non-neurological) disorders, such as Crohn’s disease, rheumatoid arthritis or psoriasis, where IL-23 is the main player—may also encourage the use of exclusive IL-12 inhibition in tackling AD.

## Methods

All animal experiments were conducted in accordance with animal welfare acts and were approved by the regional office for health and social service in Berlin (LaGeSo; license O 298/17, T 0276/07 and T-CH0022/23). Human tissue sampling, processing and subsequent analyses were done on the basis of ethical approval number EA1/144/13 granted by the Ethics Board of the Charité – Universitätsmedizin Berlin, Germany. Human postmortem CNS tissue was collected and snap frozen from patients who had given written informed consent before death. Postmortem brain tissue from the University of Florida Human Brain and Tissue Bank (UF HBTB) was collected with approval from the University of Florida Institutional Review Board (IRB201600067). All patients or their next of kin gave written informed consent for the brain donation and use of tissue specimens for research.

### Mice

Heterozygous APPPS1^+/−^ mice (previously described by Radde et al.^65^, co-expressing KM670/671NL-mutated APP and L166P-mutated presenilin 1 (PS1) under the control of a neuron-specific Thy1 promoter element, termed APPPS1 mice) were crossed to *Il12b*^−/−^ (ref. ^66^) mice and compared to WT littermate controls. Mice were bred on a C57BL/6J background. *Il12Rb2*^fl/fl^ mice, generated and characterized previously^67,68^, were crossed to APPPS1 and heterozygous Nestin^Cre^ mice^69^ (The Jackson Laboratory, stock no. 0037719), resulting in the APPPS1^+/−^.Nestin^Cre^.*Il12Rb2*^*fl/fl*^ strain. The animals used for the experiments represented in Fig. 1a,b are littermates. The genotype annotated as APPPS1 represents APPPS1^+/−^.Nestin^WT^.*Il12rb*^*WTlWT*^ with the *Il12rb2* gene still intact due to the lack of Cre recombinase expression and the lack of the LoxP site, and the genotype annotated as APPPS1.Nestin^Cre^.*Il12rb2*^*fl*/*fl*^ represents APPPS1^+/−^.Nestin^Cre^.*Il12rb2*^*fl/fl*^ animals with exon 7 excision of *Il12rb* in Nestin^+^, neuroectodermal cells (neurons, oligodendrocyte lineage cells and astrocytes). The APPPS1^+/−^.Nestin^Cre^.*Il23r*^*fl*/*fl*^ strain was generated by crossing the heterozygous APPPS1^+/−^ mouse model to the homozygous *Il23r*^*fl*/*fl*^ line^70^, where exon 4 of the *Il23r* gene is flanked by two LoxP sites, as well as the Nestin^Cre^ reporter mice. The animals used for the experiments represented in Fig. 1a,c are littermates. The genotype annotated as APPPS1 represents APPPS1^+/−^.Nestin^WT^.*Il23r*^*fl*/*fl*^ mice with the *Il23r* gene still intact due to the absence of Cre recombinase expression, and APPPS1.Nestin^Cre^.*Il23r*^*fl*/*fl*^ represents APPPS1^+/−^.Nestin^Cre^.*Il23r*^*fl*/*fl*^ mice with exon 4 excision of *Il23r* in Nestin^+^ cells.

Animals were kept in individually ventilated cages with a 12-h light/dark cycle with food and water ad libitum.

### snRNA-seq

Mouse hippocampi were harvested from male WT, APPPS1 and APPPS1.*Il12b*^−/−^ mice at the age of 250 days, immediately snap frozen in liquid nitrogen and stored at −80 °C until further processing. Nuclei were isolated from a single hippocampus in 2 ml of pre-chilled EZ PREP lysis buffer (Sigma-Aldrich, NUC-101) using a glass Dounce tissue grinder (Sigma-Aldrich, D8938) (25 strokes with pestle A and 25 strokes with pestle B), followed by incubation for 5 min on ice with an additional 2 ml of EZ PREP buffer. During incubation, 1 µM DAPI was added to the homogenate, which was then filtered through a 30 µM FACS tube filter. A BD FACSAria III flow cytometer with a 70-µm nozzle configuration was used to sort the nuclei based on the fluorescent DAPI signal at 4 °C. As CNS nuclei vary strongly in size, no doublet discrimination was performed based on forward scatter (FSC) or side scatter (SSC) to avoid bias. Nuclei were counted based on bright-field image and DAPI fluorescence using a Neubauer counting chamber and a Keyence BZX-710 microscope. Isolated nuclei were immediately used for droplet-based 3′-end single-cell RNA-seq using the Chromium Next GEM Single Cell 3′ GEM, Library & Gel Bead Kit, version 3.1, following the manufacturer’s instructions (10x Genomics, PN-1000121). The libraries were multiplexed, and three samples per lane were sequenced on an Illumina HiSeq 4000 sequencer. Demultiplexing, barcode processing, read alignment and gene expression quantification were carried out using Cell Ranger software (version 3.1.0; 10x Genomics). First, Cell Ranger mkfastq demultiplexed the sequencing by sample index. The quality of the data was checked using FastQC (version 0.11.5), and all samples showed high-quality RNA-seq data with good median per-base quality (≥28) across most of the read length. Cell Ranger count used STAR software with default parameters to align sequenced reads to the reference genome (GRCm38, Ensembl GTF, version 98). As nuclei have a high amount of pre-mRNA, we generated a custom pre-mRNA reference based with the instructions provided on the 10x Genomics website; we also included intronic reads in the final gene expression counts. Finally, the output files for all nine samples were aggregated into one gene–cell matrix using Cell Ranger ‘aggr’ without read depth normalization.

### snRNA-seq data analysis

Data were analyzed in R (version 3.6.0) using Seurat (version 3.1.2)^71^. In all downstream analyses, the filtered feature–barcode matrix was used rather than the raw feature–barcode matrix. For the initial quality control, we excluded genes expressed in fewer than three nuclei and nuclei expressing fewer than 200 genes or fewer than 500 or more than 30,000 UMIs and nuclei with more than 5% mitochondrial reads. This resulted in a dataset of 84,002 cells and 31,790 quantified genes across nine samples. We then normalized UMI counts using the ‘LogNormalize’ method and by applying a scale factor of 10,000. We found variable genes using ‘FindVariableFeatures’ with the selection method ‘vst’. Data regression was performed using the ScaleData function with nUMI, percent mitochondrial counts and sample origin as confounding factors. Dimensionality reduction was performed using principal component analysis (PCA), and we selected 40 principal components based on elbow plot. The ‘FindNeighbors’ function, which implements shared nearest neighbor (SNN) modularity optimization-based clustering algorithm, was applied and identified clusters with a resolution of 0.8 by the ‘FindClusters’ function, resulting in 45 initial clusters. A further dimensionality reduction step was carried out, using the UMAP algorithm to visualize the data. The UMAP parameters were as follows: n.neighbors = 20, min.dist = 0.35, n.epochs = 500, spread = 2. As UMAP overlay by individual sample shows minimal batch effects, we did not consider any batch correction method. For assigning clusters to cell types, we used the ‘FindAllMarkers’ function with default parameters, identifying negative and positive markers for that cluster. Scrublet (version 0.21)^72^ with an expected_doublet_rate = 0.06 was applied, resulting in detection of 5.2% of doublets. We defined doublet clusters as containing more than 50% of doublets and removed these for downstream analysis. We noticed that these clusters were projected in the middle of other cell types in UMAP and were validated as expressing marker genes from two different cell types. We eventually removed eight clusters, which reduced our dataset to 82,298 nuclei. Cell type variability was measured using an entropy-based approach^73^. We first grouped by replicate and genotype. The local neighborhood was defined by taking the 30 nearest neighbors using kNN-Graph and the relative entropy. We applied Kullback–Leibler divergence to measure how cells are distributed among samples. Controls were randomly shuffled and showed that differences detected in gene signature were of biological relevance and not driven by technical artifacts. One-way ANOVA was used to test whether cellular proportions differed by genotype. Homogeneity of variance and normality of data distribution were assessed by using Bartlett and Shapiro–Wilk tests, respectively, with the R package ‘stats’ (version 3.6.0). A *P* value of less than 0.05 was considered statistically significant. To identify differentially regulated genes among genotypes, we performed empirical Bayes quasi-likelihood *F*-tests (QLF) including the cellular detection rate (the fraction of detected genes per cell) using EdgeR (version 3.28.1)^74,75^. A log_2_ fold change (FC) greater than 0.25 and a false discovery rate (FDR) less than 0.01 were considered significant. Among differentially expressed genes, we removed the *Ttr* gene as its expression was highly dependent on the presence of a choroid plexus cluster in a given sample, suggesting a dissection bias at the stage of hippocampus isolation. GO term enrichment of each cluster was performed with topGO (version 2.36.0)^76^. In GO analysis, genes showing average log_2_FC > 0.25 and adjusted *P* < 0.01 were considered significant, and all expressed genes were used as background. We used the ‘elim’ algorithm instead of the classic method to be more conservative and excluded broad GO terms with more than 1,000 listed reference genes. We performed trajectory inference with SCORPIUS (version 1.0.7)^77^ for oligodendrocyte populations, including OPCs. To infer developmental trajectory, we used highly variable and all expressed transcriptional factor genes and reduced dimension using distance metric as Spearman with 3 number of dimensions. To infer gene expression along the pseudotime, we first downloaded a list of genes from Mouse Genome Informatics (http://www.informatics.jax.org) displaying negative regulation of oligodendrocyte differentiation (GO:0048715) and positive regulation of oligodendrocyte differentiation (GO:0048714), which was smoothed over pseudotime using a generalized additive model using ‘mgcv’ (version 1.8-28). We used CellPhoneDB (version 2.1.1)^27^ to assess cellular crosstalk between different cell types. To identify putative cell–cell interactions via multi-subunit ligand–receptor complex pairs, label permutation was performed. First, we converted mouse gene symbols to human gene symbols using biomaRt (version 2.42.1)^78^ and removed duplicated gene symbols from digital gene expression matrix. We then calculated normalized data with scale factor 10,000. Finally, we conducted statistical analyses by randomly permuting the cluster labels of each cell 10,000 times.

A list of DAM genes was downloaded from the work by Keren-Shaul et al.^33^. This DAM signature was collected from single-cell sorting and downstream single-cell RNA-seq of microglia from the AD mouse model 5×FAD. We computed the log_2_FCs of the Microglia 3 (Disease-associated cluster) to Microglia 1 (Homeostatic cluster) ratio for each gene^33^, resulting in 461 DAM genes. To identify DAM APPPS1-related signature genes from our dataset, we compared the differential expression of cluster 8 (disease state) and cluster 3 (homeostatic state), where only logFC > 0.25 and FDR < 0.01 were considered, ultimately resulting in 488 genes. Ninety-six genes intersected between our study and the already published study^33^. Of those, 365 genes were specific for the published DAM signature, whereas 392 genes were specific to our APPPS1-related dataset.

To visualize read coverage of snRNA-seq data in a genome browser, Sambamba (version 0.6.8)^79^ was used to sort BAM file produced from 10x Cell Ranger count. We extracted only primary alignment reads from sorted BAM file and created a bedgraph file using bedtools (version 2.27.1)^80^ with normalized using read depth and split file by strand specific. Finally, we created a BigWig file using bedGraphToBigWig (version 4)^81^, and the resulting genomic signal tracks were visualized in the UCSC Genome Browser. The results of genome tracks are located in public hubs at the MDC Genome web browser (https://genome.mdc-berlin.de/).

To validate our results derived from analyzing human postmortem brain tissue (Fig. 3f), we downloaded gene matrix and meta data of primary motor cortex from https://portal.brain-map.org/ (ref. ^82^). Like previous analyses using Seurat (version 4), we filtered genes expressed in fewer than three nuclei. We then normalized UMI counts and found variable genes using ‘vst’ and kept 2,000 highly variable genes (HVGs). We scaled data to perform PCA and selected 40 principal components for downstream analyses, FindNeighbor, FindCluster and RunUMAP. Cell types were assigned by information from meta data.

### Bulk RNA-seq and analysis

Total RNA was isolated from freshly frozen hippocampi from 250-day-old animals using a NucleoSpin miRNA and RNA purification kit (Macherey Nagel, 740971.50). The tissue was homogenized using a Pellet Mixer (VWR, 431-0100) in 0.35 ml of Buffer ML provided in the RNA purification kit and subsequently passing the homogenate through a 23-gauge needle (B. Braun, 465 7667) until no clumps remained. RNA was isolated according to the manufacturer’s protocol and eluted in 20 µl of RNAse-free water. Library construction and bulk mRNA-seq were performed by Novogen (non-stranded cDNA libraries; 150-bp paired-end run with a depth of 40 million reads per library). Bulk transcriptomes were aligned using STAR (version 2.7.1a)^83^ with mm10 reference and quantified using featureCounts (version 1.6.0). Differential expression genes between samples were determined as adjusted *P* value less than 0.05 and FC higher than 1 or lower than −1 using DESeq2 (version 1.24.0)^84^, using default option without the lfcShrink function. To analyze pairwise correlations between bulk transcriptomes and snRNA-seq data, bulk transcriptomes were converted to transcripts per million (TPM) by dividing each count of each gene by its length and multiplying by 1 million. Each gene length was calculated from GTFtools (version 0.6.9)^85^ by median length of its isoforms. snRNA-seq expression counts were summed by each sample and converted to counts per million (CPM). The scale of all figures is log_2_ (CPM/TPM + 1). To validate the findings provided by bulk transcriptomics regarding the ratio of various CNS cell types, we performed weighted non-negative least squares for cell type proportion estimation using Multi-subject Single Cell deconvolution (MuSiC) (version 0.1.1)^16^. We ran the package with default settings with HVGs from snRNA-seq data.

### Multiplex smFISH

Frozen brain tissue was placed in a tissue mold (Sakura, SA62534-15) and submerged in Tissue-Tek freezing medium (Sakura, 4583). Then, 10-µm-thick tissue sections were cut using a cryostat (Thermo Fisher Scientific, HM 560), placed on SuperFrost Plus slides (R. Langenbrink, 500621) and dried for 1 h at −20 °C. Tissue processing for RNAscope multiplex staining (Advanced Cell Diagnostics) was done following the manufacturer’s protocol for fresh-frozen sections. In brief, tissue was fixed in freshly prepared 4% paraformaldehyde (PFA) (pH 7.4) for 30 min at 4 °C, followed by alcohol dehydration. Tissue was exposed at room temperature to the given concentration of H_2_O_2_ for 10 min and to Protease IV (Bio-Techne, 322340) for 30 min and then incubated for 2 h with target probes at 40 °C in a HybEZ Hybridization System (Bio-Techne, 321711). The following target probes were used: Mm-Il12rb1 (Bio-Techne, 488761), Mm-Il12rb2 (Bio-Techne, 451301), Mm-Il23r (Bio-Techne, 403751), Mm-Aldh1l1-C2 (Bio-Techne, 405891-C2), Mm-Slc1a3-C2 (Bio-Techne, 430781-C2), Mm-Gfap-C2 (Bio-Techne, 313211-C2), Mm-Sox10-C2 (Bio-Techne, 435931-C2), Mm-Tmem119-C3 (Bio-Techne, 472901-C3), Mm-Sall1-C3 (Bio-Techne, 469661-C3), Mm-Rbfox3-C3 (Bio-Techne, 313311-C3) and Mm-Map2-C3 (Bio-Techne, 431151-C3). Signal amplification was achieved using an RNAscope Multiplex Fluorescent Kit, version 2 (Bio-Techne, 323110), closely following the manufacturer’s protocol. Probes were labeled with Opal 520 (1:500; C2 probe, FP1487001KT; PerkinElmer), Opal 570 (1:500; C1 probe, FP1488001KT; PerkinElmer) and Opal 690 (1:500; C3 probe, FP1497001KT; PerkinElmer), and three-dimensional image stacks (1 µm step size, ×40 objective) of stained sections were taken on a Leica TCS SP5 confocal laser scanning microscope using an HCX PL APO lambda blue ×63 oil UV objective controlled by LAS AF scan software (Leica Microsystems).

### Electron microscopy imaging

Tissue processing and electron microscopy imaging were performed as described previously^86^. In brief, animals were perfused with 10 ml of HBSS (Gibco, 24020117) followed by perfusion with 30 ml of Karlsson Schultz buffer, pH 7.4 (4% formaldehyde, 2.5% glutaraldehyde EM grade, 0.5% NaCl in phosphate buffer, pH 7.4)^87^. After perfusion, brains were further fixed in Karlsson Schultz buffer for an additional 24 h at 4 °C, followed by storage in 1% PFA in phosphate buffer, pH 7.4, until further processing. Then, 200-μm coronal sections of brains were cut using a Leica VT1200S vibratome. The caudal part of the corpus callosum, including the alveus as well as the fimbria, was punched out of the section using a 1-mm Harris Uni-core Punch and embedded in Epon (Serva) after post-fixation with 2% OsO_4_ (Science Services) and dehydration using acetone. Epon-embedded samples were cut using a microtome (Leica, UC7). Semi-thin sections were collected to verify the region of interest (ROI) using a light microscope. Ultra-thin 50-nm sections were placed on hexagonal copper grids (Gilder) coated with ‘Formvar’ (Plano) and stained with Uranyless (Science Services). Ultra-thin sections were analyzed using a LEO912 electron microscope (Zeiss), and pictures were obtained using a wide-angle dual-speed 2K-CCD camera (TRS) at ×5,000 magnification. To quantitatively compare the axons between the groups, the electron microscopy images were analyzed using ImageJ. To avoid bias, all image analyses were done blinded, and a grid was put onto each image, and those axons that touched the angles were measured, in total 20 axons per image. The axon areas were measured using the free-hand tool to outline the outer layer of the axoplasm membrane. To account for myelin thickness, the inner tongue of the myelin sheath was measured as well as the outer layer, which was also used for axon diameter calculation. The axon and fiber diameters were calculated from the measured axon area using the formula 2 × √ A/π. Axon sheath thickness was then calculated by subtracting the axon diameter from the fiber diameter. The g-ratio was calculated by dividing the axon diameter by the fiber diameter. The data were tested for normality using the Shapiro–Wilk test. Differences in g-ratios of the observed groups were compared and tested with the Kruskal–Wallis test for non-parametric data in multiple groups. The results were adjusted using the Bonferroni correction for multiple testing.

### Immunohistochemical stainings, quantitative assessments and microglial phagocytosis assay

Animals were perfused with PBS, and hemispheres were fixed for 24 h in 4% PFA at 4 °C. Brains were further processed by incubating them in 10%, 20% and, finally, 30% sucrose in PBS over the course of 3 days. Free-floating brain sections were cut at 30–40-µm thickness using a cryostat (Thermo Fisher Scientific, NX70 957030L) and stored in cryoprotectant (0.65 g of NaH_2_PO_4_ × H_2_O, 2.8 g of Na_2_HPO_4_ in 250 ml of ddH_2_O, pH 7.4, with 150 ml of ethylene glycol and 125 ml of glycerine) at 4 °C until further use. Sections were stained by first incubating them in 0.3% Triton-X in PBS with 10% normal goat serum for 1 h. The primary antibodies used for detecting oligodendrocytes were Olig2 (rabbit, 1:750, AB9610; Millipore), CC1 (mouse, 1:200, OP80-100UG; Merck) and MBP (rat, 1:200, MCA409S; Bio-Rad); PV (rabbit, 1:200, MAB1572; Millipore) for depicting interneurons; Clec7a (rat, 1:150, mapg-mdect; InvivoGen) and Iba1 (rabbit, 1:500, 019-19741; Wako) for identifying microglia; and 4G8 (mouse, 1:1;000, SIG39320; Covance) for visualizing Aβ plaques, and all were incubated at 4 °C overnight. The secondary antibodies Alexa Fluor 568 goat anti-rabbit (A11011), Alexa Fluor 488 goat anti-mouse IgG (A11001), Alexa Fluor 488 goat anti-mouse IgG2b (A21141), Alexa Fluor 647 goat anti-rabbit (A21244), all Invitrogen; Cy3 donkey anti-rat IgG (Jackson ImmunoResearch, 712-165-153); and Alexa Fluor 488 goat anti-rat (112-545-003, 1:300; Dianova) were added for 2 h at room temperature. Nuclei were counterstained using 500 nM DAPI for 1 min.

For assessment of oligodendrocytes, 3–6 brain sections were stained and imaged with an inverted microscope (Leica, DMI 6000) at ×40 magnification. Images were stitched together automatically using LAS X 3.3 Stage Experiment Tilescan software (Leica). Based on the DAPI-stained image, the hippocampus was set as the ROI. Based on a fixed threshold for all images, ImageJ was applied to quantify all DAPI as well as Olig2^+^ nuclei using the ‘Analyze Particle’ function. The total count of Olig2^+^ nuclei was normalized to the total counts of DAPI^+^ nuclei. For technical reasons, CC1^+^ cells were counted manually with the Cell Counter tool in ImageJ and normalized to Olig2^+^ cells. Similarly, cell bodies of PV^+^ interneurons were counted using the Cell Counter tool in ImageJ within a tissue area of 2 × 10^4^ µm^2^ in the CA1 and within a tissue area of 3 × 10^4^ µm^2^in the cortex. Images were taken with an inverted Nikon spinning disk confocal microscope at a magnification of ×10. Three distinct but equally located ROIs were analyzed per tissue section. The mean gray value of MBP in the corpus callosum was calculated via binarization based on a threshold within the defined ROI and normalized to DAPI mean gray value of the same image. The MBP-covered area in the cortex was measured with the ‘Analyze Particles’ tool in ImageJ. Methods were adapted from published data^88^.

Preparation of acute cortical brain slices from 90-day-old *Il12b*^*+/+*^, *Il12b*^−/−^, APPPS1.*Il12b*^*+/+*^ and APPPS1.*Il12b*^−/−^ mice followed previously described protocols^89^: mice were decapitated, and brains were removed and washed in artificial CSF (aCSF), containing 134 mM NaCl, 2.5 mM KCl, 1.3 mM MgCl_2_, 2 mM CaCl_2_, 1.25 mM K_2_HPO_4_, 26 mM NaHCO_3_ and 10 nM D-glucose, pH 7.4, which was saturated with carbogen (95% O_2_, 5% CO_2_). Coronal cortical slices at 130-µm thickness were prepared at 4 °C using a vibratome and were then kept in aCSF at room temperature for 2 h until phagocytosis experiment. The microglial phagocytosis assay in acute brain slices was done as described previously^89^. In brief, yellowgreen fluorescent Fluoresbrite carboxylated microspheres (2 µm diameter; Polysciences Europe) were coated with FCS by shaking at 100*g* for 30 min at room temperature. Microspheres were centrifuged at 1,000*g* for 5 min and then washed twice in HBSS and applied on acute brain slices at 8.4 × 10^6^ microspheres per well. Slices were incubated for 1 h at 37 °C. Afterwards, they were washed twice with 1× PBS on an orbital shaker for 15 min and then fixed with 4% PFA for 1 h at room temperature. To prepare the tissue for analyzing microglia phagocytosis, fixed slices were permeabilized in 2% TX in PBS for 4 h at room temperature on a shaker, and then unspecific binding sites were blocked by incubation in 10% NGS/2%TX/2% BSA for 2 h at room temperature. Primary antibody Iba1 was diluted at 1:300 in 5% NGS/0.3% TX in PBS and incubated with slices overnight at 4 °C. The next day, slices were washed three times in 1× PBS, and secondary antibody (goat anti-rabbit Alexa Fluor 568, 1:300, in PBS/5% NGS/0.3% TX) was incubated for 2 h at room temperature. After washing slices three times with 1× PBS, they were counterstained with Draq5 (1:1,000 in PBS; eBioscience) for 10 min and then mounted with Microscopy Aquatex (Merck). For assessing microglial phagocytosis, 15-µm-thick z-stacks with a step size of 1 µm, beginning from the top of the slice, where the microspheres are located, were taken at ×40 magnification using a confocal laser scanning microscope (Zeiss, LSM 510 META). Four to five z-stacks per slice were analyzed by determining the percentage of phagocytic microglia per field of view using the ImageJ Cell Counter plugin. Furthermore, phagocytic cells were grouped according to the number of microspheres they had taken up, to determine the phagocytosis grade of microglia, with 1–3 microspheres = grade 1; 4–6 = grade 2; 7–10 = grade 3; and more than 10 = grade 4.

To assess the expression of Clec7a by microglia in tissue sections of brains of mice, image stacks were taken with an inverted Nikon spinning disk confocal microscope at a magnification of ×20. Three distinct but equally located ROIs were analyzed per section with 12 sections per animal. Clec7a expression levels of plaque-associated microglia (Fig. 7h) were quantified from maximum projections of the confocal stacks. The quantification was performed in an automated manner using custom-written ImageJ macros (segmentation)^90^ and Python scripts (statistics and distance measurements), which can be found on GitHub^91^. The data displayed in the histogram were binned image-wise, normalized (divided by their own integral) and then pooled by calculating the median from all images per animal and plotting the mean and s.e.m. of all animals from one group. DAPI signal was de-noised (frequency filter in Fourier space for structures above 2.6 µm) and blurred (Gaussian blur, sigma = 520 nm) before using StarDist^92^ (‘versatile fluorescent nuclei model’) to segment nuclei. Watershed segmentation was then applied to the Euclidean distance map of the binary images to separate merged nuclei. Plaques were segmented from the de-noised 4G8 channel (frequency filter in Fourier space for structures above 26 µm), followed by ‘rolling ball’ background subtraction (radius = 52 µm) and Otsu binarization. Only objects above 130 µm^2^ were regarded as plaques. The mean intensities within segmented nuclear regions (s. above) were used as a measure for Iba1 and Clec7a expression levels. Cells were classified as Iba1 positive/negative by auto-thresholding (Otsu’s method on all cell-specific expression levels within one image). Only Iba1^+^ cells within 30 µm around plaques were used for analysis.

### Mouse brain tissue protein extraction and quantification

Brain tissue used for protein extraction was harvested from PBS-perfused animals. Snap-frozen proteins were extracted based on their solubility as previously described^93^: thawed hemispheres with removed cerebellum were homogenized successively in TBS buffer pH 7.6 (20 mM Tris, 137 mM NaCl), Triton-X buffer (TBS buffer supplemented with 1% Triton X-100) and SDS buffer (2% SDS in ddH_2_O). All buffers were kept on ice and supplemented with cOmplete Mini Protease Inhibitor Cocktail Tablets (Roche, 11836153001). Initial homogenization was performed mechanically using a tissue homogenizer (VWR, 431-0100), followed by passing the homogenate through a 2-ml syringe with a G25 cannula (BD Microlance, 03086976). TBS and Triton-X brain extracts were incubated for 30 min on ice, and SDS homogenate was incubated at room temperature and vortexed every 5 min. After incubation, the homogenate was centrifuged at 100,000*g* for 45 min at 4 °C. The supernatant was collected, snap frozen in liquid nitrogen and stored at 80 °C until further use. The residual pellet was re-suspended in succeeding buffers. Total protein concentration was quantified via QuantiPro BCA Protein Assay Kit (Pierce) according to the manufacturerʼs protocol using an Infinite M200 photometer (Tecan). White and gray matter were isolated from the corpus callosum and cortex of mouse brains and homogenized in PBS, and the protein concentration was adjusted by BCA assay to 1 mg ml^−1^ protein before its use for mass spectrometry. To assess Aβ levels, the 96-well MultiSpot Human 6E10 Aβ Triplex Assay Kit (Meso Scale Diagnostics (MSD)) was used as described previously^94^: the plate was blocked by adding Diluent 35 (provided by the manufacturer) for 60 min, followed by a wash with 0.05% Tween 20 in PBS, pH 7.6. TBS and Triton-X fractions were used undiluted, whereas the SDS fraction was diluted 1:500 in Diluent 35. Each sample was added to a well supplemented with a detection antibody solution containing 2% 50× SULFO-TAG 6E10 detection antibody and 1% Aβ40 blocker in Diluent 100. The plate was read using an MS6000 machine (MSD). For murine brain tissue, TBS, Triton-X and SDS fractions were analyzed, whereas, for human tissue, TX and SDS fractions were assessed.

### Lipidomics

Compound extraction was performed using a mixture of methanol, methyl tert-butyl ether and chloroform (MMC, 4:3:3, v/v/v)^95^. The MMC solvent was supplemented with SPLASH LIPIDOMIX standard and additional internal standards, d7-sphinganine (SPH d18:0), d7-sphingosine (SPH d18:1), dihydroceramide (Cer d18:0/12:0), ceramide (Cer d18:1/12:0), deoxydihydroceramide (Cer m18:0 12:0) deoxyceramide (Cer m18:1 12:0) and glucosylceramides (GluCer d18:1/8:0 and GlcCer d18:1 18:1 (d5)) (Avanti Polar Lipids). Liquid chromatography was carried out as described previously^96^ with some modifications. Lipids were separated using a C30 Accucore LC column (150 mm × 2.1 mm, 2.6-µm particle size) and a Transcend UHPLC pump (Thermo Fisher Scientific). Mass spectrometry analysis was done on a hybrid quadrupole-orbitrap mass spectrometer (Thermo Fisher Scientific, Q-Exactive). Mass spectra were acquired with a resolution accuracy of 5 ppm from predicted masses at a resolving power of 70,000 at 200 *m*/*z*. Positive and negative ionization modes were acquired alternately. Data analysis was performed using Compound Discoverer 3.3.2.31 (Thermo Fisher Scientific) for retention time alignment, peak picking, annotation and matching to the metabolite databases LipidBlast VS68 and Metlin Experimental Mass Spectral Database, version 2017 (Scripps Center for Metabolomics)^96,97^. The web-based platform MetaboAnalyst (https://www.metaboanalyst.ca) was used to analyze the lipidomics data. With the integrated Statistical Analysis module, heatmaps and partial least squares-discriminant analysis (PLS-DA) were generated and visualized.

### Human postmortem CNS tissue gene and protein expression analyses

Patients with AD were stratified by the ‘ABC’ score for AD neuropathology as defined by Montine et al.^98^. Hippocampal tissue was homogenized using a Pellet Mixer (VWR, 431-0100) in 0.30 ml of RLT lysis buffer provided in the RNA purification kit (Qiagen, 74004) and subsequently passing the homogenate through a 23-gauge needle (B. Braun, 465 766) until no clumps remained. RNA was isolated according to the manufacturer’s protocol and eluted in 18 µl of RNAse-free water. cDNA was generated using a High-Capacity cDNA Reverse Transcription Kit (Thermo Fisher Scientific, 4368813). qPCR was performed by TaqMan Fast Universal Master (Thermo Fisher Scientific, 4364103) and primers *Il12rb* (Thermo Fisher Scientific, Hs00155486_m1), *Il23r* (Thermo Fisher Scientific, Hs00332759_m1) and GAPDH (Thermo Fisher Scientific, Hs02786624_g1) on QuantStudio 6 Flex (Applied Biosystems) were used. Gene transcriptional level was analyzed using CT values by the ΔΔCT method^99^. Human protein extraction was performed in the same fashion as for mouse brain tissue (see above). For IL12p70 measurements, the soluble TBS fraction was used, and IL12p70 was measured using a human IL12p70-specific high-sensitivity ELISA (Thermo Fisher Scientific, BMS238HS) according to the manufacturer’s instructions.

### Myelinating mouse spinal cord and primary oligodendrocyte cell cultures

Oligodendrocytes and neurofilaments were cultured from embryonic day 13 (E13) mouse spinal cords as described by Thomson et al.^100^. Cultures were supplemented with insulin until 12 days in vitro (DIV12), followed by adding IL-12p70 (10 ng ml^−1^, recombinant murine IL-12 p70, 210-12; PeproTech), IL-12p80 (5 ng ml^−1^, recombinant murine IL-12 p80, 210-12P80H; PeproTech) or vehicle to the medium for the indicated duration. Primary oligodendrocytes were sorted via magnetic-activated cell sorting (MACS) using O4 beads and cultivated for 5 days until treatment with either IL-12p70 (10 ng ml^−1^, recombinant murine IL-12 p70, 210-12; PeproTech) or IL-12p80 (5 ng ml^−1^, recombinant murine IL-12 p80, 210-12P80H; PeproTech). Myelinating cultures were harvested at DIV30. Cells on coverslips were fixed with 4% PFA for 10 min and washed with PBS before permeabilization with 0.5% Triton-X in PBS. Unspecific binding was blocked using 10% normal goat serum in PBS, and primary antibodies recognizing MBP (rat, MCA409S, 1:200; Bio-Rad) and neurofilament (mouse, 837904, 1:500; BioLegend) were added and incubated overnight at 4 °C. Secondary antibodies (Alexa Fluor 488 goat anti-rat, 112-545-003, 1:300; Dianova and Alexa Fluor 647 donkey anti-mouse, A31571, 1:300; Invitrogen) were added and incubated for 1 h at room temperature. Cells were counterstained with DAPI as described above (see ‘Immunohistochemical stainings, quantitative assessment and microglial phagocytosis assay’ subsection).

### Human oligodendrocyte-like cell cultures

The human oligodendroglioma cell line (Sigma-Aldrich, SCC163) was cultured in growth medium (GM) (DMEM, high-glucose D6546-6X500ML, Thermo Fisher Scientific; 10% FCS, 1% penicillin–streptomycin and 1% L-glutamin). For differentiation, 100,000 cells per six wells were plated in differentiation medium (DM) (DMEM (D6546-6X500ML, Thermo Fisher Scientific), 0.05% FCS, 1% penicillin–streptomycin, 1% Insulin-Transferrin-Selenium (ITS-G) (Thermo Fisher Scientific, 41400045) and 30 nM 3,3′,5-triiod-l-thyronin (Merck, T-074-1ML)) for 2 days. As a control, 100,000 cells per six wells were plated in GM. After 2 days, DM was supplemented with 10 ng ml^−1^ FGF-2 and 10 ng ml^−1^ PDGF-AA and matured for an additional day while cells in GM were fed with GM. After 3 days, cells grown in GM reached 100% confluency, whereas cells grown in DM were at approximately 30% confluency. Next, oligodendrocytes were stimulated in GM with 85 nM or 1.5 nM IL-12p70, IL-12p80 or IL-23 dissolved in 0.25% BSA in PBS for 24 h as depicted in Extended Data Fig. 5e–i. Control wells (vehicle-treated) were stimulated with 0.25% BSA in PBS. For qPCR, cells were harvested, and RNA was isolated using the Qiagen miRNA kit according to the manufacturer’s instructions. MBP qPCR was performed by TaqMan Fast Universal Master (Thermo Fisher Scientific, 4364103) using primers MBP (Thermo Fisher Scientific, Hs00921945_m1) and GAPDH (Thermo Fisher Scientific, Hs02786624_g1) on QuantStudio 6 Flex (Applied Biosystems). Gene transcriptional levels were analyzed using CT values by the ΔΔCT method^99^. Cytokines were measured using a V-Plex Cytokine Panel 1 Human Kit (MSD, K15959D-1) on an MS6000 machine (MSD).

### Statistics and reproducibility

As for in vitro data, at least two independent experiments were performed with at least three technical replicates per condition. Sample sizes were chosen based on previous experiments, and no statistical method was used to predetermine sample size for all experiments. For immunohistochemical stainings, an *n* of 6 per genotype was chosen; for biochemical (Meso Scale) as well as lipidomics analyses, an *n* of 8 was chosen. For snRNA-seq analyses an *n* of 3 per genotype was taken. The investigators were blinded during image acquisition and analysis. All data were checked for normal distribution before choosing the appropriate statistical test.

For the identification of differentially regulated gene expression in snRNA-seq data, we removed the *Ttr* gene as its expression was highly dependent on the presence of a choroid plexus cluster in a given sample, suggesting a bias due to the dissection at the stage of hippocampus isolation (indicated in the Methods section).

When performing the 6E10 Aβ Triplex Assay using protein extraction generated from the brains of APPPS1.NestinCre.*Il23r*^*fl/fl*^ animals, a few readings generated the output ‘NaN’ due to a technical error. The individual data points, which represented non-available values, were excluded in subsequent analyses and explains the fewer data points in Fig. 1e.

### Reporting summary

Further information on research design is available in the Nature Portfolio Reporting Summary linked to this article.

## Supplementary information

Reporting Summary

Supplementary Table 1 Human postmortem tissue information.

Supplementary Table 2 Common genes upregulated in snRNA-seq of APPPS1 and 5×FAD mice (Keren-Shaul et al.^33^).

Supplementary Table 3 Genes upregulated in microglia of APPPS1 mice (snRNA-seq data).

## Source data

Source Data Fig. 5 Unprocessed western blots and quantification.

## Data Availability

The raw sequencing and processed data are available in the Gene Expression Omnibus under accession number GSE173242, and a shiny app was created, which allows access to single-nucleus data for any gene of interest interactively via the following URL: https://shiny.mdc-berlin.de/AD_Neuroinflammation/. Lipidomics data for re-analysis are available in a Zenodo repository at 10.5281/zenodo.14620944. Source data and immunohistochemical image files will be provided upon reasonable request.

## Code availability

Codes for snRNA-seq data are available at https://github.com/m21camby/AD_Neuroinflammation. Codes for microglia workflow are available at https://github.com/ngimber/AlzheimersWorkflow.
