# Supplementary material for: Interleukin-12 signaling drives Alzheimer’s disease pathology through disrupting neuronal and oligodendrocyte homeostasis
Source: Nat Aging. 2025 Mar 13;5(4):622–41. doi: 10.1038/s43587-025-00816-2 (PMC12003168; doi:10.1038/s43587-025-00816-2)
Supplement: Supplementary file 1 — Reporting Summary [file 43587_2025_816_MOESM1_ESM.pdf]

nature portfolio

Corresponding author(s): Frank Heppner

Last updated by author(s): Dec 20, 2024

Reporting Summary

Nature Portfolio wishes to improve the reproducibility of the work that we publish. This form provides structure for consistency and transparency in reporting. For further information on Nature Portfolio policies, see our Editorial Policies and the Editorial Policy Checklist.

Statistics

For all statistical analyses, confirm that the following items are present in the figure legend, table legend, main text, or Methods section.

n/a	Confirmed	
☐	☒	The exact sample size (*n*) for each experimental group/condition, given as a discrete number and unit of measurement
☒	☐	A statement on whether measurements were taken from distinct samples or whether the same sample was measured repeatedly
☐	☒	The statistical test(s) used AND whether they are one- or two-sided<br>*Only common tests should be described solely by name; describe more complex techniques in the Methods section.*
☒	☐	A description of all covariates tested
☐	☒	A description of any assumptions or corrections, such as tests of normality and adjustment for multiple comparisons
☐	☒	A full description of the statistical parameters including central tendency (e.g. means) or other basic estimates (e.g. regression coefficient) AND variation (e.g. standard deviation) or associated estimates of uncertainty (e.g. confidence intervals)
☐	☒	For null hypothesis testing, the test statistic (e.g. *F*, *t*, *r*) with confidence intervals, effect sizes, degrees of freedom and *P* value noted<br>*Give P values as exact values whenever suitable.*
☒	☐	For Bayesian analysis, information on the choice of priors and Markov chain Monte Carlo settings
☒	☐	For hierarchical and complex designs, identification of the appropriate level for tests and full reporting of outcomes
☒	☐	Estimates of effect sizes (e.g. Cohen's *d*, Pearson's *r*), indicating how they were calculated

Our web collection on statistics for biologists contains articles on many of the points above.

Software and code

Policy information about availability of computer code

Data collection	snRNA-seq data were generated/collected using Chromium Next GEM Single Cell 3′ GEM, Library & Gel Bead Kit v3.1 and sequenced on an Illumina HiSeq 4000 sequencer. Demultiplexing, barcode processing, read alignment and gene expression quantification was carried out using Cell Ranger software (v3.1.0, 10x Genomics). Confocal images were collected using LAS X 3.3 Stage Experiment Tilescan software (Leica) and Nikon Spinning Disk Confocal microscope. EM (ultrastructural) images were taken at LEO912 Electron-Microscope (Zeiss). Lipidomic data were generated using a hybrid quadrupole-Orbitrap mass spectrometer (Q-Exactive, Thermo Fisher Scientific). qPCR was performed on QuantStudio™ 6 Flex (Applied Biosystems). MSD data were generated using a MS6000 machine (MSD).
Data analysis	Computational data was analyzed in R (v3.6.0) using Seurat (v3.1.2), Scrublet (v0.21), R package stats (v3.6.0), EdgeR (v3.28.1), topGO (v2.36.0), SCORPIUS (v1.0.7), mgcv (v1.8-28), CellPhoneDB (V2.1.1), biomaRt (v2.42.1), Sambamba (v0.6.8), bedtools (v2.27.1), bedGraphToBigWig (v4), GTFtools(v0.6.9). Images were analyzed using Image J (v1.52). Further data anylsis were done using GraphPad Prism (v9). Analysis of mass spectrometry data was performed using Compound Discoverer 3.3.2.31 (Thermo Fisher Scientific) and matched to metabolite databases LipidBlast VS68 and Metlin Experimental Mass Spectral Database Vers 2017 (Scripps Center for Metabolomics). MetaboAnalyst 6.0 (https://metaboanalyst.ca) was used to plot lipidomics data.

For manuscripts utilizing custom algorithms or software that are central to the research but not yet described in published literature, software must be made available to editors and reviewers. We strongly encourage code deposition in a community repository (e.g. GitHub). See the Nature Portfolio guidelines for submitting code & software for further information.

Data

Policy information about availability of data

All manuscripts must include a data availability statement. This statement should provide the following information, where applicable:

- Accession codes, unique identifiers, or web links for publicly available datasets
- A description of any restrictions on data availability
- For clinical datasets or third party data, please ensure that the statement adheres to our policy

The raw sequencing and processed data is available in GEO under the accession GSE173242 and a shiny app was created, which allows to access single-nuclei data for any gene of interest interactively via the following URL: https://shiny.mdc-berlin.de/AD_Neuroinflammation/. Lipidomics data for re-analysis are available on Zenodo repository under the DOI: 105281/zenodo.14620944. Source data and immunhistological image files will be provided upon request.

Field-specific reporting

Please select the one below that is the best fit for your research. If you are not sure, read the appropriate sections before making your selection.

☒ Life sciences ☐ Behavioural & social sciences ☐ Ecological, evolutionary & environmental sciences

For a reference copy of the document with all sections, see nature.com/documents/nr-reporting-summary-flat.pdf

Life sciences study design

All studies must disclose on these points even when the disclosure is negative.

Sample size	If data were gained to be used as an exploratory approach, n=3 per genotype was choosen in order to be able to perform basic statistical analyses (snRNA-seq, EM imaging). For biochemical and molecular analyses, n=5 or n=9 were choosen to account for known biological variability. For lipidomics, we used an n of 8 due to the same biological and technical reasons. For immunohistological stainings we used an n of 6 to 8 per genotype and n=3-12 slices depending on the experiment which we have applied from experience in other studies. For cell culture experiments, we have run five independent experiments each with three technical replicates per condition. We have not statistically calculated sample sizes prior to experimental design, but chosen these numbers with our best knowledge.
Data exclusions	For the identification of differentially regulated gene expression in snRNA-seq data, we removed the Ttr gene as its expression was highly dependent on the presence of a Choroid Plexus cluster in a given sample, suggesting a dissection bias at the stage of hippocampus isolation (indicated in the Methods Section).<br>Performing the 6e10 MSD assay on protein extraction generated from brain tissue of APPPS1.NestinCre.Il23Rfl/fl animals, a few readings generated the output "NaN" due to technical error. This individual data point as a non-available value was therefore excluded in the subsequent analysis. This is indicated by the fewer data points in Figure 1e.
Replication	Cell culture experiments were repeated with similar outcome in five independent experiments. Stainings and all molecular analyses have not been repeated due to the value of the tissue except for the MBP staining in Fig. 4f to compensate for variablity in individual animals , but results are aligned between one another and when comparing young and old animals. We have made sure to approach the different aspects (such as myelination) in as many independent analyses as possible.
Randomization	Animals allocated to experimental groups were based on genotype. No method of randomization has been applied.
Blinding	Investigators were blinded for tissue collection and processing as well as subsequent data collection (e.g., microscopy) and analysis. Additionally, different investigators were involved in different analyses and the data was later combined, resulting in an objective that convinces us of the validity of our results.

Reporting for specific materials, systems and methods

We require information from authors about some types of materials, experimental systems and methods used in many studies. Here, indicate whether each material, system or method listed is relevant to your study. If you are not sure if a list item applies to your research, read the appropriate section before selecting a response.

Materials & experimental systems

n/a	Involved in the study	
☐	☒	Antibodies
☐	☒	Eukaryotic cell lines
☒	☐	Palaeontology and archaeology
☐	☒	Animals and other organisms
☐	☒	Human research participants
☒	☐	Clinical data
☒	☐	Dual use research of concern

Methods

n/a	Involved in the study	
☒	☐	ChIP-seq
☒	☐	Flow cytometry
☒	☐	MRI-based neuroimaging

Antibodies

Antibodies used

Olig2 (Rabbit, 1:750, AB9610, Milipore), Anti-APC clone CC-1 (Mouse, 1:200, OP80-100UG, Merck), MBP clone 12 (Rat, 1:200, MCA409S, Biorad), Parvalbumin clone PARV-19 (Rabbit, 1:200, MAB1572, Millipore), Clec7a clone R1-8g7 (Rat, 1:150, mapg-mdect, InvivoGen), Iba1 (Rabbit, 1:500, 019-19741, Wako), 4G8 residues 17-24 of Abeta (Mouse, 1:1000, SIG39320, Covance), Alexa568 goat anti rabbit (A11011; 1:300, Invtitrogen), Alexa488 goat anti-mouse IgG (A11001; 1:300, Invitrogen), Alexa488 goat anti-mouse IgG2b (A21141, 1:300, Invitrogen), Alexa647 goat anti-rabbit (A21244, 1:300, Invitrogen), Cy3 donkey anti-rat IgG (712-165-153, 1:300, Jackson ImmunoResearch), and Alexa488 goat anti-rat (112-545-003, 1:300, Dianova)

Validation

Olig2: Evaluated by immunohistochemistry on glioblastoma by vendor. Immunohistochemistry (Paraffin):
Representative lot data.Anti-Olig2 (AB9610) staining pattern morphology in glioblastoma. Tissue was pretreated with TE Buffer, pH 9.0. Polyclonal antibody was diluted to 1:500, using IHC-Select® Detection with HRP-DAB. Optimal Staining With TE Buffer Epitope Retrieval: Glioblastoma
Anti-APC Antibody, clone CC-1 detects levels of APC proteins & has been published & validated for use in IHC. Immunohistochemistry Analysis: A representative lot from an independent laboratory detected APC in human brain tissue containing multiple schlerosis lesions (Saikali, P. et al. (2007). J Neurosci. 27(5):1220-1228.).
Immunohistochemistry Analysis: A representative lot from an independent laboratory detected APC in rat spinal cord injury tissue (McTique, D. M., et al. (2001). J Neurosci. 21(10):3392-3400.).
Immunohistochemistry Analysis: A 1:5 dilution from a representative lot detected APC in human colorectal cancer tissue, human smooth muscle tissue, and human colon tissue.

MBP clone 12: This product has been reported to work in the following applications: ELISA, immunofluorescence, radioimmunoassays, Western Blotting. This information is derived from testing within our laboratories, peer-reviewed publications or personal communications from the originators.
Relvas, J.B. et al. (2001) Expression of dominant-negative and chimeric subunits reveals an essential role for beta1 integrin during myelination. Curr Biol. 11: 1039-43.

Parvalbumin clone PARV-19 has been used in immunofluorescence:
A Ca(2+)-binding protein with numerous roles and uses: parvalbumin in molecular biology and physiology.
BioEssays : news and reviews in molecular, cellular and developmental biology (2009-03-11)
Syed Hasan Arif
PMID19274659

4G8: co-localisation with pFTAA stained amyloid beta plaques (M. Jendrach, unpublished data); no signal in wild type microglia (T. Pugeda, unpublished data)
This antibody is reactive to amino acid residues 17-24 of ß amyloid. The epitope lies within amino acids 18-22 of ß amyloid (VFFAE).
4G8 ß-amyloid antibody reacts to abnormally processed isoforms, as well as precursor forms.
This antibody clone has been reported for use on IHC of free-floating sections in PBS containing 1% Triton incubated with 0.1 m citrate buffer (4).
Poduslo JF, et al. 2004. Biochem. 43:6064. (IHC-F) PubMed
Forny-Germano L, et al. 2014. J Neurosci. 34:13629. (IHC-Other) PubMed
Vallino Costassa E, et al. 2016. J Alzheimers Dis. 51: 875:87. (IHC-P) PubMed
Chen X, et al. 2013. Neurobiol Aging. 34:2370. (ICC) PubMed
Hatami A, et al. 2016. J Alzheimers Dis. 50:517. (IHC-P) PubMed

Iba1: standard antibody to visualize microglia (4732 citations): "Fujifilm Wako's "Anti Iba1, Rabbit (for immunocytochemistry)" (Product Number 019-19741), which allows even microglia processes to be stained by immunohistochemical staining, is used by researchers all over the world as a microglia marker antibody standard."
Imai, Y., Ibata, I., Ito, D., Ohsawa, K. & Kohsaka, S.: Biochemical and biophysical research communications, 224(3), 855(1996).
A Novel Geneiba1 in the Major Histocompatibility Complex Class III Region Encoding an EF Hand Protein Expressed in a Monocytic Lineage
Mori, I., Imai, Y., Kohsaka, S. & Kimura, Y.: Microbiology and immunology, 44(8), 729(2000).
Upregulated expression of Iba1 molecules in the central nervous system of mice in response to neurovirulent influenza A virus infection
Sasaki, Y., Ohsawa, K., Kanazawa, H., Kohsaka, S. & Imai, Y.: Biochemical and biophysical research communications, 286(2), 292(2001).
Iba1 is an actin-cross-linking protein in macrophages/microglia.
Zhao, S. et al.: Cell, 180(4), 796(2020).
Cellular and Molecular Probing of Intact Human Organs
Ahn, J.H. et al.: Lab. Anim. Res., 28(3), 165 (2012).
Comparison of alpha-synuclein immunoreactivity in the spinal cord between the adult and aged beagle dog
Ide, T. et al.: J. Vet. Med .Sci., 72(1), 99 (2010).
Histiocytic Sarcoma in the Brain of a Cat
Gaige, S. et al.: Neurotoxicology, 34, 135(2013).
c-Fos immunoreactivity in the pig brain following deoxynivalenol intoxication: Focus on NUCB2/nesfatin-1 expressing neurons
Rodriguez-Callejas, J.D. et al.: Front. Aging Neurosci., 8, 315(2016).
Evidence of Tau Hyperphosphorylation and Dystrophic Microglia in the Common Marmoset
Fantin, A. et al.: Blood, 116(5), 829(2010).
Tissue macrophages act as cellular chaperones for vascular anastomosis downstream of VEGF-mediated endothelial tip cell induction

Clec7a: supplier: "binding of Anti-mDectin-1-IgG to mDectin-1 on cells has been validated using flow cytometry". Co-stainings with Iba1 in amyloid beta plaque-associated microglia (manuscript and EMBO Rep 2020 21(3):e48530), no signal in wild type microglia (M. Jendrach, unpublished data)
clone R1-8g7: This product has been validated for neutralization using cellular assays.
Binding of Anti-mDectin-1-IgG to mDectin-1 on cells has been validated using flow cytometry.

The absence of bacterial contamination (e.g. lipoproteins and endotoxins) has been confirmed using HEK-Blue™ TLR2 and HEK-Blue™ TLR4 cells.
1. Fischer M. et al., 2017. Isoform localization of Dectin-1 regulates the signaling quality of anti-fungal immunity. Eur J Immunol. 47(5):848-859.
2. Hou H. et al., 2017. C-type Lectin Receptor: Old Friend and New Player. Med Chem. 13(6):536-543.
3. Drummond RA. & Lionakis MS., 2016. Mechanistic Insights into the Role of C-Type Lectin Receptor/ CARD9 Signaling in Human Antifungal Immunity. Front Cell Infect Microbiol. 6:39.
4. Romero MM. et al., 2016. Reactive oxygen species production by human dendritic cells involves TLR2 and dectin-1 and is essential for efficient immune response against Mycobacteria. Cell Microbiol. 18(6):875-86.

beta-Actin: standard antibody to visualize Actin (4100 citations); no staining of mouse heart tissue (M. Jendrach, unpublished data)

Eukaryotic cell lines

Policy information about cell lines

Cell line source(s)	The human oligodendroglioma cell line (SCC163, Sigma)
Authentication	Directly obtained from vendor (Sigma), therefore only tested within our experimental set-up (regular control experiments, see respective figure).
Mycoplasma contamination	The cell line was not tested for mycoplasma contamination.
Commonly misidentified lines (See ICLAC register)	No commonly misidentified cell lines were used in this study.

Animals and other organisms

Policy information about studies involving animals; ARRIVE guidelines recommended for reporting animal research

Laboratory animals	Mus musculus, C57BL/6. Mice for snRNA-seq study: male, 250-day-old. Mice for immunohistochemistry, EM imaging, biochemical and molecular analysis: 120 and 250-day-old, mixed sexes.<br>APPPS1.NestinCre.Il12Rb2fl/fl: 250 day old. Mice for Mesoscale analysis. Mixed sexes.<br>APPPS1.NestinCre.Il23Rfl/fl: 250-day-old. Mice for Mesoscale analysis. Mixed sexes.<br>APPPS1.Il12b: 120 and 250-day-old, mixed sexes, used for lipidomics, eletronmicroscopy, immunohistochemistry.<br>C57BL/6: E13, used for myelinating cell culture experiments.<br>C57BL/6: P3 used for MACS-sorting of oligodendrocytes.
Wild animals	This study did not involve wild animals.
Field-collected samples	This study did not involve samples collected from the field.
Ethics oversight	All animal experiments were conducted in accordance with animal welfare acts and were approved by the regional office for health and social service in Berlin (LaGeSo; license O 298/17, T 0276/07 and T-CH0022/23)

Note that full information on the approval of the study protocol must also be provided in the manuscript.

Human research participants

Policy information about studies involving human research participants

Population characteristics	Postmortem tissue was collected from participants with neurological diseases as indicated, based on a written informed
Recruitment	Subjects recruited provided written informed consent prior to death.
Ethics oversight	Human tissue sampling, processing and subsequent analyses were done on the basis to the ethical approval Nr. EA1/144/13 granted by the Ethics Board of the Charité – Universitätsmedizin Berlin, Germany. Postmortem brain tissue from the University of Florida Human Brain and Tissue Bank (UF HBTB) was collected with approval from the University of Florida Institutional Review Board (IRB201600067). All the patients or their next-of-kin gave written informed consent for the brain donation and use of tissue specimens for research.

Note that full information on the approval of the study protocol must also be provided in the manuscript.
